# Six new species of zombie-ant fungi from Yunnan in China

**DOI:** 10.1186/s43008-023-00114-9

**Published:** 2023-05-11

**Authors:** Dexiang Tang, Ou Huang, Weiqiu Zou, Yuanbing Wang, Yao Wang, Quanying Dong, Tao Sun, Gang Yang, Hong Yu

**Affiliations:** 1 Yunnan Herbal Laboratory, College of Ecology and Environmental Sciences, Yunnan University, Kunming, 650504 China; 2 School of Life Science, Yunnan University, Kunming, 650504 China; 3 Yunnan Key Laboratory for Fungal Diversity and Green Development, Kunming Institute of Botany, Chinese Academy of Sciences, Kunming, 650201 China; 4 The Council of Management and Conservation of Sun River National Park, Puer, 665000 China

**Keywords:** 6 new taxa, *Camponotus*, Living cultures, Morphology, Multi-gene phylogeny, *Ophiocordyceps*, *Polyrhachis*, Taxonomy

## Abstract

Some *Ophiocordyceps* species infecting ants are able to manipulate the host behavior. The hosts are manipulated in order to move to location that are advantageous for fungal spore transmission. *Ophiocordyceps* species that are able to manipulate the ant's behavior are called "zombie-ant fungi". They are widespread within tropical forests worldwide, with relatively few reports from subtropical monsoon evergreen broad-leaf forest. Zombie-ant fungi have been described and reported in different countries worldwide. However, there were a few reports from China. This study proposed six new species of zombie-ant fungi from China based on multi-gene (SSU, LSU, *TEF*, *RPB1* and *RPB2*) phylogenetic analyses and morphological characteristics. Six novel species of *Ophiocordyceps* from China were identified as the *Ophiocordyceps unilateralis* core clade, forming a separate lineage with other species. Six novel species of *Ophiocordyceps* with hirsutella-like asexual morphs exclusively infecting ants were presented herein, namely, *Ophiocordyceps acroasca*, *Ophiocordyceps bifertilis*, *Ophiocordyceps subtiliphialida*, *Ophiocordyceps basiasca*, *Ophiocordyceps nuozhaduensis* and *Ophiocordyceps contiispora*. Descriptions and illustrations for six taxon were provided. Five of these species were collected from the subtropical monsoon evergreen broad-leaf forest, and one was collected from the rainforest and subtropical monsoon evergreen broad-leaf forest. This work proposes that the same host of *Camponotus* can be infected by multiple ant pathogenic fungi, while multiple ants of *Polyrhachis* can be infected by the same pathogenic fungi at the same time. This study contributes towards a better understanding of the evolutionary relationship between hosts and fungi, and provides novel insights into the morphology, distribution, parasitism, and ecology of *Ophiocordyceps unilateralis *sensu lato. We have provided a method for obtaining living cultures of *Ophiocordyceps unilateralis* complex species and their asexual morphs based on the living cultures, which is of significant value for further studies of *Ophiocordyceps unilateralis* complex species in the future.

## INTRODUCTION

Evolutionary relationships between fungi and insects, from parasitism to mutualism, have been widely studied (Suh et al. 2005; Cheek et al. 2020; Haelewaters et al. 2020). Insects are diverse, with more than a million described species (Foottit and Adler 2009), in 29 orders (Misof et al. 2014). The fungal pathogens are able to colonize 19 of 29 orders, resulting in the evolution of extensive diversity of strategies and morphologies, by using the insect body for infection and onward transmission (Araújo and Hughes 2016). Among these insects and fungi strategies, one of the most impressive and sophisticated involved ants and species of fungi within the genus *Ophiocordyceps* (Andersen et al. 2009). The species of *Ophiocordyceps* had colonized 13 orders of insects (Crous et al. 2004; Araújo and Hughes 2016), comprised of more than 300 species of entomopathogens (Kepler et al. 2011; Sanjuan et al. 2015; Crous et al. 2016; Araújo et al. 2018; Khonsanit et al. 2018; Araújo and Hughes 2019; Wei et al. 2020; Tang et al. 2022; Xu et al. 2022). The insect hosts orders infected by these fungi included Coleoptera, Diptera, Hemiptera, Hymenoptera, Isoptera, Lepidoptera, Neuroptera, Dragonflies, and Orthoptera (Araújo et al. 2015; Araújo and Hughes 2019). Ants (Hymenoptera) were widely distributed in the arctic to tropical, occupying a wide range of habitats from high canopy to leaf litter; their colonies ranged from a few dozen (Jahyny et al. 2002) to millions of individuals (Currie et al. 2003). In tropical forests, they contributed as much as 50% of animal biomass (Hölldobler et al. 2009). Among the hosts of many entomopathogenic fungi, ants were also the most common host of species within *Ophiocordyceps* (Evans and Samson 1982; Evans et al. 2011b; Kepler et al. 2011; Luangsa-ard et al. 2011; Kobmoo et al. 2012, 2015; Araújo et al. 2015, 2018, 2020; Sanjuan et al. 2015; Spatafora et al. 2015; Crous et al. 2016; Tasanathai et al. 2019; Wei et al. 2020; Tang et al. 2022; Xu et al. 2022).

*Ophiocordyceps* was erected by Petch (1931) to accommodate the species of *Cordyceps* that produce non-disarticulating ascospores. The term as a subgeneric classification was used by Kobayasi, based solely on ascospores morphology, and essentially adopted the diagnosis of Petch (Kobayasi 1941; Petch 1931). Then the subgenera *Ophiocordyceps* was transferred as subgenus of *Cordyceps *sensu lato (Mains 1958). The three new families were well-supported in Sung et al. (2007) study, hence their proposition to split them into 3 families (Ophiocordycipitaceae, Clavicipitaceae and Cordycipitaceae). *Ophiocordyceps* was proposed as a genus of Ophiocordycipitaceae. The classification system of *Cordyceps *sensu lato was widely accepted (Sung et al. 2007). *Ophiocordyceps unilateralis *sensu stricto was originally published as *Torrubia unilateralis* (Tulasne and Tulasne 1865). *Torrubia unilateralis* was transferred to *Ophiocordyceps* (Petch 1931). Evans et al. (2018) moved to epitypify *O. unilateralis *sensu stricto and to clarify its description, providing an interpretive type that was more effective in a biological sense than the illustrations by Tulasne; it was proposed to distinguish *O. unilateralis *sensu stricto and *O. unilateralis *sensu lato. Asexual morphs associated with *Ophiocordyceps* included *Hirsutella*, *Syngliocladium*, *Stilbella*, *Paraisaria*, *Hymenostilbe* and *Sorosporella* (Quandt et al. 2014). *Hirsutella*, *Stilbella*, *Paraisaria* and *Hymenostilbe* were recorded to be associated with ants. Asexual morphs *Hymenostilbe* and *Hirsutella* were commonly found associated with ants (Evans and Samson 1982, 1984; Araújo et al. 2015; Araújo and Hughes 2017).

Members of the *O. unilateralis* complex were ordinary among the pathogenic fungi on ants (Evans et al. 2011a, 2011b). These fungi could change ant behavior controlling it to leave the nest to die, usually in an exposed position in which they were attached or biting leaves or branches in a "death grip" (Hughes et al. 2011). The manipulative behavior caused by species within *O. unilateralis* complex has attracted extensive attention (Moore 1995, Thomas et al. 2010, Poulin and Maure 2015, de Bekker et al. 2018, Hafer-Hahmann 2019, Will et al. 2020). However, the mechanism of manipulating host behavior remained unknown (Herbison 2017; Will et al. 2020). Many studies have often used the term *O. unilateralis *sensu lato for the zombie-ant fungus, including the evolutionary relationship between fungi and hosts, the mechanism of manipulating host behavior, and genomes (Andersen et al. 2009; Hughes et al. 2009; Pontoppidan et al. 2009; Evans et al. 2011a). Regarding the evolutionary relationship between fungi and hosts, Evans et al. (2011b) found that different fungi parasitized different ants; their appearances were very similar but differed in morphological characters. A total of thirty-six species of the *O. unilateralis *sensu lato have been described. Although this group was estimated to be tens or even hundreds of species worldwide (Evans et al. 2011a), or 580 species discussed by Araújo et al. (Araújo et al. 2018, Araújo and Hughes, unpublished data). There are many species of *O. unilateralis *sensu lato need further global collections to provide more new taxa to support for exploring the evolutionary relationship between the fungus and its host.

Previous some taxonomic works supported the “one ant-one *Ophiocordyceps* species” hypothesis (Evans et al. 2011b; Kobmoo et al. 2012; Araújo et al. 2018). They pointed out that host-specific fungal species seemed to be associated to each ant species, leading to the "one ant-one fungus", and the host identity was used as a proxy for fungal identification, such as *O. camponoti-atricipis*, *O. camponoti-balzani*, *O. camponoti-bispinosi*, *O. camponoti-chartificis*, *O. camponoti-femorati*, *O. camponoti-floridani*, *O. camponoti-hippocrepidis*, *O. camponoti-indiani*, *O. camponoti-leonardi*, *O. camponoti-melanotici*, *O. camponoti-nidulantis*, *O. camponoti-novogranadensis*, *O. camponoti-renggeri*, *O. camponoti-rufipedis*, *O. camponoti-saundersi*, *O. camponoti-sexguttati*, and *O. polyrhachis-furca* (Evans et al. 2011b; Kobmoo et al. 2012; Araújo et al. 2015, 2018). However, with the deepening of research, different views have emerged, two hosts of the *genus Polyrhachis* were infected by the ant pathogenic fungus "*O. nooreniae*" (Crous et al. 2016). Lin et al. (2020) showed that a single species of *O. unilateralis *sensu lato can infect eight ant species. In addition, Kobmoo et al. (2019) indicated that the ant pathogenic fungus may parasitize the same host based on population genomics study, and constitute further cryptic species, challenging the one ant-one fungus paradigm. The relationship between *O. unilateralis *sensu lato complex and Formicine ants is still uncertain. Host identification was an important feature to describe and report new taxa. However, in our research, observing hundreds of specimens, we identified that some vital characteristics of the host (such as mouthparts, antennae, legs and abdomens) have been destroyed by pathogenic fungi. Therefore, constructing a host phylogenetic tree using molecular data (*COI* genes) is of great significance to explore the evolutionary relationship between host and species of *O. unilateralis *sensu lato.

*Ophiocordyceps unilateralis *sensu lato has been described and reported in the past two decades. Eighteen species were described from Brazil (Evans and Samson 1982; Evans et al. 2011b; Araújo et al. 2015, 2018), one from Colombia (Araújo et al. 2018), three from the USA (Araújo et al. 2018), one from Ghana (Spatafora et al. 2015), three from Australia (Crous et al. 2016; Araújo et al. 2018), three from Japan (Kepler et al. 2011; Araújo et al. 2018), six from Thailand (Luangsa-ard et al. 2011; Kobmoo et al. 2012, 2015), one from China (Wei et al. 2020). In the past three years, we have also found the species of *O. unilateralis *sensu lato in Laos and Vietnam (unpublished data). Although multiple taxa of *O. unilateralis *sensu lato have been described, many questions remain open within the group, such as the evolutionary relationship between host and *O. unilateralis *sensu lato species, the origins of the group, and the mechanisms that manipulate host behavior. The description and record of the new taxa of *O. unilateralis *sensu lato is of great importance for the solution of the above problems.

Most species of *O. unilateralis *sensu lato have been collected from tropical rainforests. There are few or no record of *O. unilateralis *sensu lato species in the subtropical monsoon evergreen broad-leaf forest. Few species of *O. unilateralis *sensu lato were reported in China (Wei et al. 2020). The unique geographical location of southwest China is an important area for the diversity of *Cordyceps *sensu lato. Many species of *Ophiocordyceps* have been reported from Yunnan province, for example, *O. laojunshanensis* (Chen et al. 2011), *O. lanpingensis* (Chen et al. 2013), *O. alboperitheciata* (Fan et al. 2021), *O. pingbianensis* (Chen et al. 2021). Our team has spent the past more than two decades investigating and collecting entomopathogenic fungi to describe more new species and to solve taxonomic problems. The six novel species presented herein were collected from Yunnan province in China. Based on morphological and phylogenetic analyses, all species were identified as part of the core clade of *O. unilateralis*. This study aims to provide additional new taxa that support understanding of the evolutionary relationships between fungi and their hosts, providing novel insights into their living cultures, morphology, ecology, parasitism, and distribution.

## MATERIALS AND METHODS

### Sampling and isolation

All specimens were collected from Yunnan Province in China in this work. Most specimens were collected from Sun River National Park; some were from Nuozhadu Nature Reserve and Mohan Town, Mengla County. Specimens were noted (e.g., vegetation type, death position, altitude above ground) and photographed in the field, then placed in a sterilized boxes, returned to the laboratory, and stored at 4 °C. Before obtaining axenic cultures, the specimens' fertile region (ascomata) was examined using an Olympus SZ61 stereomicroscope (Olympus Corporation, Tokyo, Japan). Stromata was removed from the head of the ant for morphological observation (sexual and asexual morph). The sclerotium (body of the ant) was immersed in 30% H_2_O_2_ for 5–8 min, immersed in 75% ethanol for 1 min, and rinsed five times in sterilized water (the specimens must be complete). After drying on sterilized filter paper, the sclerotium was divided into four segments (the head and abdomen were divided into the same two-part, respectively) and inoculated onto solid medium plates (potato 200 g/L, dextrose 20 g/L, agar 20 g/L, yeast powder 10 g/L and peptone 5 g/L), cultured at 25–28 °C (normal temperature was the best condition). Pure cultures were transplanted to a PDA slant, and stored at 4 °C. The specimens were deposited in the Yunnan Herbal Herbarium (YHH) of Yunnan University. The cultures were stored in Yunnan the Fungal Culture Collection (YFCC) of Yunnan University.

### Morphological observations

For sexual morph observation, ascomata were photographed and measured by using an Olympus SZ61 stereomicroscope (Olympus Corporation, Tokyo, Japan). Free-hand or frozen sections of the fruiting structures were mounted in lactophenol cotton blue solution for microscopic study and photomicrography. The frozen sections were used by Freezing Microtome HM525NX (Thermo Fisher Scientific, Massachusetts, America). Micro-morphological characteristics (perithecia, asci, apical caps and ascospores) of fungi were examined using Olympus CX40 and BX53 microscopes. Two methods were used for asexual morphological observations. One was directly observed from stromata, sutures, legs and joints of specimens, and another was observed from the pure culture on solid medium plates. Cultures on solid medium plates were incubated for 30–40 days at 25 °C and photographed using a Canon 750 D camera (Canon Inc., Tokyo, Japan). The solid medium was made 0.5–1 mm thick, then divided into 5 mm long and 5 mm wide. Finally, the medium was placed on the glass slide in the sterile culture dish (there was a glass rod to cushion that it could not be submerged in sterile water). The colony was placed on a solid medium, gently covered the cover slide, added sterile water 3 ml, and placed at 25 °C for 30–40 days. The BX53 microscope and Olympus CX40 were used to examine the asexual characteristics such as conidiophores, conidiogenous cells and conidia. Unfortunately, we were not able to study the germination process in most species because the samples had been previously dried.

### DNA extraction, polymerase chain reaction (PCR), and sequencing

Specimens and axenic living cultures were prepared for DNA extraction, and the specimens were treated in the same way as the axenic cultures prior to DNA extraction. Total DNA was extracted using the CTAB method, following the described by Liu et al. (2001). Five genes (SSU, LSU, *TEF*, *RPB1*, *RPB2*) and *COI* genes were amplified and sequenced. The primer pair NS1 and NS4 were used to amplify a fraction of the nuclear ribosomal small subunit (SSU) (White et al. 1990). The primer pair LR0R (Hopple 1994) and LR5 (Vilgalys and Hester 1990) were used to amplify the nuclear ribosomal large subunit (LSU). The primer pair 2218R and 983F were used to amplify the translation elongation factor 1α (*TEF*) (Rehner and Buckley 2005). The primer pairs RPB1 and RPB1Cr_oph, fRPB2-7cR and fRPB2-5F, were used to amplify the largest and second largest subunits of RNA polymerase II (*RPB1* and *RPB2*), respectively (Liu et al. 1999; Castlebury et al. 2004; Araújo et al. 2018). The primer pair, LCO1490 and HCO2198 (Hebert et al. 2003) was used to amplify the *COI* gene. The polymerase chain reaction (PCR) matrix was performed in a final volume of 25 µl, composed of 17.25 µl of sterile water, 2.5 µl of PCR 10 × Buffer (2 mmol/l Mg2+) (Transgen Biotech, Beijing, China), 2 µl of dNTP (2.5 mmol/l), 1 µL of forwarding primers (10 µmol/), 1 µl of reverse primers (10 µmol/l), 0.25 µl of Taq DNA polymerase (Transgen Biotech, Beijing, China), 1 µl of DNA template (500 ng/µl). Amplification reactions were performed in a BIO-RAD T100TM thermal cycler (BIO-RAD Laboratories, Hercules, CA, United States). The PCR program of five genes was conducted as described by Wang et al. (2020), and the *COI* gene was conducted as described by Hebert et al. (2003). The Beijing Genomics Institute (Chongqing, China) performed the target gene amplification and sequencing.

### Phylogenetic analyses

#### Phylogenetic analyses of fungi

Phylogenetic analyses were based on sequences of five genes (SSU, LSU, *TEF*, *RPB1* and *RPB2*). Sequences of multiple genes from various species (see Table 1) were retrieved from GenBank and the nucleotide sequences were combined with those generated in our study. Information on specimens and GenBank accession numbers were listed in Table 1. Sequences were aligned using Clustal X (v.2.0) (Larkin et al. 2007), poorly-aligned regions were removed and adjusted manually using MEGA6 (v.6.0) (Tamura et al. 2013). We generated one fungi dataset (SSU, LSU, *TEF*, *RPB1* and *RPB2*). Modelfinder (Kalyaanamoorthy et al. 2017) was used to select the best-fitting likelihood model for maximum likelihood (ML) analyses, and Bayesian inference (BI) analyses were carried out for the fungi datasets. The Corrected Akaike Information Criterion (AIC) was used to select the model for each gene, and the best-fitting models were provided in Table 3. For ML analyses, tree searches were performed in IQ-tree (v.2.1.3) (Nguyen et al. 2015) based on the best-fit model with 5000 ultrafast bootstraps (Hoang et al. 2017) in a single run. BI analyses were conducted using MrBayes (v.3.2.2) (Ronquist et al. 2012). Four Markov Chain Monte Carlo chains were run, each beginning with a random tree and sampling, one tree every 100 generations of 2000,000 generations, and the first 25% of samples were discarded as burn-in. Each tree was visualized with its maximum-likelihood bootstrap support values (ML-BS) and Bayesian inference posterior probability (BI-PP) in Figtree (v.1.4.3). Adobe Illustrator CS6 was used for editing.

**Table 1:** Voucher information, GenBank accession numbers, host and location of the taxa used in this study

Species	Voucher information	SSU	LSU	*TEF*	*RPB1*	*RPB2*	Host	Location
*Hirsutella* sp.	NHJ 12525	EF469125	EF469078	EF469063	EF469092	EF469111	Hemiptera	–
*Hirsutella* sp.	OSC 128575	EF469126	EF469079	EF469064	EF469093	EF469110	Hemiptera	–
*Ophiocordyceps acicularis*	ARSEF 5692	DQ522540	DQ518754	DQ522322	DQ522368	DQ522418	Coleoptera	Korea
***Ophiocordyceps acroasca***	**YFCC 9049**	**ON555837**	**ON555918**	**ON567757**	**ON568677**	**ON568130**	***Camponotus* sp.**	**China**
***Ophiocordyceps acroasca***	**YFCC 9019**	**ON555838**	**ON555919**	**ON567758**	**ON568678**	**ON568131**	***Camponotus* sp.**	**China**
***Ophiocordyceps acroasca***	**YFCC 9017**	**ON555839**	**ON555920**	**ON567759**	**ON568679**	**ON568132**	***Camponotus* sp.**	**China**
***Ophiocordyceps acroasca***	**YFCC 9018**	**ON555840**	**ON555921**	**ON567760**	**ON568680**	**ON568133**	***Camponotus* sp.**	**China**
***Ophiocordyceps acroasca***	**YFCC 9016**^T^	**ON555841**	**ON555922**	**ON567761**	**ON568681**	**ON568134**	***Camponotus* sp.**	**China**
***Ophiocordyceps acroasca***	**YHH 20122**	**ON555842**	**–**	**ON567762**	**ON568682**	**–**	***Camponotus* sp.**	**China**
*Ophiocordyceps albacongiuae*	RC20	KX713633	**–**	KX713670	–	**–**	*Camponotus* sp.	Colombia
*Ophiocordyceps annullata*	CEM 303	KJ878915	KJ878881	KJ878962	KJ878995	**–**	Coleoptera	Japan
*Ophiocordyceps aphodii*	ARSEF 5498	DQ522541	DQ518755	DQ522323	**–**	DQ522419	Coleoptera	**–**
*Ophiocordyceps australis*	HUA 186097	KC610786	KC610765	KC610735	KF658662	**–**	Hymenoptera	Colombia
***Ophiocordyceps basiasca***	**YHH 20191**	**ON555828**	**ON555910**	**ON567748**	**ON568672**	**ON568121**	***Camponotus* sp.**	**China**
***Ophiocordyceps bifertilis***	**YFCC 9012**^T^	**ON555843**	**ON555923**	**ON567763**	**ON568143**	**ON568135**	***Polyrhachis* sp.**	**China**
***Ophiocordyceps bifertilis***	**YHH 20162**	**ON555844**	**–**	**ON567764**	**ON568144**	**–**	***Polyrhachis* sp.**	**China**
***Ophiocordyceps bifertilis***	**YHH 20163**	**ON555845**	**ON555924**	**ON567765**	**ON568145**	**ON568136**	***Polyrhachis* sp.**	**China**
***Ophiocordyceps bifertilis***	**YHH 20164**	**ON555846**	**–**	**ON567766**	**ON568146**	**–**	***Polyrhachis* sp.**	**China**
***Ophiocordyceps bifertilis***	**YFCC 9048**	**ON555847**	**ON555925**	**ON567767**	**ON568147**	**ON568137**	***Polyrhachis* sp.**	**China**
***Ophiocordyceps bifertilis***	**YFCC 9013**	**ON555848**	**ON555926**	**ON567768**	**ON568148**	**ON568138**	***Polyrhachis* sp.**	**China**
*Ophiocordyceps blakebarnesii*	MISSOU5	KX713641	KX713610	KX713688	KX713716	**–**	*Camponotus* sp.	USA
*Ophiocordyceps blakebarnesii*	MISSOU4	KX713642	KX713609	KX713685	KX713715	**–**	*Camponotus* sp.	USA
*Ophiocordyceps brunneipunctata*	OSC 128576	DQ522542	DQ518756	DQ522324	DQ522369	DQ522420	Coleoptera	–
*Ophiocordyceps buquetii*	HMAS_199617	KJ878940	KJ878905	KJ878985	KJ879020	**–**	Hymenoptera	China
*Ophiocordyceps camponoti-balzani*	G143	KX713658	KX713595	KX713690	KX713705	**–**	*Camponotus balzani*	Brazil
*Ophiocordyceps camponoti-balzani*	G104	KX713660	KX713593	KX713689	KX713703	**–**	*Camponotus balzani*	Brazil
*Ophiocordyceps camponoti-bispinosi*	OBIS5	KX713636	KX713616	KX713693	KX713721	**–**	*Camponotus bispinosus*	Brazil
*Ophiocordyceps camponoti-bispinosi*	OBIS4	KX713637	KX713615	KX713692	KX713720	**–**	*Camponotus bispinosus*	Brazil
*Ophiocordyceps camponoti-chartificis*	MF080	MK874744	–	MK863824	–	**–**	*Camponotus chartifex*	Brazil
*Ophiocordyceps camponoti-femorati*	FEMO2	KX713663	KX713590	KX713678	KX713702	**–**	*Camponotus femoratus*	Brazil
*Ophiocordyceps camponoti-floridani*	Flo4	KX713662	KX713591	–	–	**–**	*Camponotus femoratus*	Brazil
*Ophiocordyceps camponoti-floridani*	Flx2	–	KX713592	KX713674	–	**–**	*Camponotus femoratus*	Brazil
*Ophiocordyceps camponoti-hippocrepidis*	HIPPOC	KX713655	KX713597	KX713673	KX713707	**–**	*Camponotus hippocrepis*	Brazil
*Ophiocordyceps camponoti-indiani*	INDI2	KX713654	KX713598	–	–	**–**	*Camponotus indianus*	Brazil
*Ophiocordyceps camponoti-leonardi*	C27	–	–	JN819019	–	**–**	*Camponotus leonardi*	Thailand
*Ophiocordyceps camponoti-leonardi*	C25	–	–	JN819029	–	**–**	*Camponotus leonardi*	Thailand
*Ophiocordyceps camponoti-nidulantis*	NIDUL2	KX713640	KX713611	KX713669	KX713717	**–**	*Camponotus nidulans*	Brazil
*Ophiocordyceps camponoti-novogranadensis*	Mal63	KX713648	KX713603	–	–	**–**	*Camponotus novogranadensis*	Brazil
*Ophiocordyceps camponoti-novogranadensis*	Mal4	KX713649	KX713602	–	–	**–**	*Camponotus novogranadensis*	Brazil
*Ophiocordyceps camponoti-renggeri*	RENG2	KX713632	–	KX713672	–	**–**	*Camponotus renggeri*	Brazil
*Ophiocordyceps camponoti-renggeri*	ORENG	KX713634	KX713617	KX713671	–	**–**	*Camponotus renggeri*	Brazil
*Ophiocordyceps camponoti-rufipedis*	G177	KX713657	KX713596	KX713680	–	**–**	*Camponotus rufipes*	Brazil
*Ophiocordyceps camponoti-rufipedis*	G108	KX713659	KX713594	KX713679	KX713704	**–**	*Camponotus rufipes*	Brazil
*Ophiocordyceps camponoti-saundersi*	C40	KJ201519	–	JN819012	–	**–**	*Camponotus saundersi*	Thailand
*Ophiocordyceps camponoti-saundersi*	Co19	–	–	JN819018	–	**–**	*Camponotus saundersi*	Thailand
*Ophiocordyceps citrina*	TNSF 18537	–	KJ878903	KJ878983	–	KJ878954	Hemiptera	Japan
*Ophiocordyceps clavata*	CEM 1762	KJ878916	KJ878882	KJ878963	KJ878996	**–**	Coleoptera	China
*Ophiocordyceps cochlidiicola*	HMAS_199612	KJ878917	KJ878884	KJ878965	KJ878998	**–**	Lepidoptera	China
***Ophiocordyceps contiispora***	**YFCC 9025**	**ON555829**	**ON555911**	**ON567749**	**ON568139**	**ON568122**	***Camponotus* sp.**	**China**
***Ophiocordyceps contiispora***	**YHH 20145**	**ON555830**	**-**	**ON567750**	**ON568140**	**ON568123**	***Camponotus* sp.**	**China**
***Ophiocordyceps contiispora***	**YFCC 9026**	**ON555831**	**ON555912**	**ON567751**	**ON568141**	**ON568124**	***Camponotus* sp.**	**China**
***Ophiocordyceps contiispora***	**YFCC 9027**^T^	**ON555832**	**ON555913**	**ON567752**	**ON568142**	**ON568125**	***Camponotus* sp.**	**China**
*Ophiocordyceps curculionum*	OSC 151910	KJ878918	KJ878885	–	KJ878999	**–**	Coleoptera	Guyana
*Ophiocordyceps daceti*	MF01	–	KX713604	KX713667	–	**–**	*Daceton armigerum*	Brazil
*Ophiocordyceps dipterigena*	OSC 151911	KJ878919	KJ878886	KJ878966	KJ879000	**–**	Diptera	USA
*Ophiocordyceps dipterigena*	OSC 151912	KJ878920	KJ878887	KJ878967	KJ879001	**–**	Diptera	USA
*Ophiocordyceps formicarum*	TNSF 18565	KJ878921	KJ878888	KJ878968	KJ879002	KJ878946	Hymenoptera	Japan
*Ophiocordyceps formosana*	TNMF 13893	KJ878908	–	KJ878956	KJ878988	KJ878943	Coleoptera	Taiwan
*Ophiocordyceps forquignonii*	OSC 151902	KJ878912	KJ878876	–	KJ878991	KJ878945	Diptera	France
*Ophiocordyceps forquignonii*	OSC 151908	KJ878922	KJ878889	–	KJ879003	KJ878947	Diptera	France
*Ophiocordyceps ghanensis*	Gh41	KX713656	–	KX713668	KX713706	**–**	*Polyrhachis* sp.	Ghana
*Ophiocordyceps halabalaensis*	MY1308^T^	KM655825	–	GU797109	–	**–**	*Camponotus gigus*	Thailand
*Ophiocordyceps halabalaensis*	MY5151	KM655826	–	GU797110	–	**–**	*Camponotus gigas*	Thailand
*Ophiocordyceps irangiensis*	OSC 128577	DQ522546	DQ518760	DQ522329	DQ522374	DQ522427	Hymenoptera	–
*Ophiocordyceps irangiensis*	OSC 128579	EF469123	EF469076	EF469060	EF469089	EF469107	Hymenoptera	–
*Ophiocordyceps kimflemingiae*	SC30	KX713629	KX713622	KX713699	KX713727	**–**	*Camponotus castaneus*/*americanus*	USA
*Ophiocordyceps kimflemingiae*	SC09B	KX713631	KX713620	KX713698	KX713724	**–**	*Camponotus castaneus*/*americanus*	USA
*Ophiocordyceps kniphofioides*	HUA 186148	KC610790	KF658679	KC610739	KF658667	KC610717	Hymenoptera	Colombia
*Ophiocordyceps konnoana*	EFCC 7295	EF468958	–	–	EF468862	EF468915	Coleoptera	Korea
*Ophiocordyceps konnoana*	EFCC 7315	EF468959	–	EF468753	EF468861	EF468916	Coleoptera	Korea
*Ophiocordyceps lloydii*	OSC 151913	KJ878924	KJ878891	KJ878970	KJ879004	KJ878948	Hymenoptera	Ecuador
*Ophiocordyceps longissima*	TNSF 18448	KJ878925	KJ878892	KJ878971	KJ879005	**–**	Hemiptera	Japan
*Ophiocordyceps longissima*	HMAS_199600	KJ878926	–	KJ878972	KJ879006	KJ878949	Hemiptera	China
*Ophiocordyceps melolonthae*	OSC 110993	DQ522548	DQ518762	DQ522331	DQ522376	**–**	Coleoptera	–
*Ophiocordyceps melolonthae*	Ophgrc 679	–	KC610768	KC610744	KF658666	**–**	Coleoptera	Colombia
*Ophiocordyceps monacidis*	MF74C	KX713646	KX713606	–	–	–	*Dolichoderus bispinosus*	Bazil
*Ophiocordyceps monacidis*	MF74	KX713647	KX713605	–	KX713712	**–**	*Dolichoderus bispinosus*	Brazil
*Ophiocordyceps myrmecophila*	CEM 1710	KJ878928	KJ878894	KJ878974	KJ879008	**–**	Hymenoptera	China
*Ophiocordyceps naomipierceae*	DAWKSANT	KX713664	KX713589	–	KX713701	**–**	*Polyrhachis* cf.* robsonii*	Australia
*Ophiocordyceps neovolkiana*	OSC 151903	KJ878930	KJ878896	KJ878976	KJ879010	**–**	Coleoptera	Japan
*Ophiocordyceps nigrella*	EFCC 9247	EF468963	EF468818	EF468758	EF468866	EF468920	–	Korea
*Ophiocordyceps nooreniae*	BRIP 55363^T^	NG065096	NG059720	KX673812	–	KX673809	*Chariomyrma* cf. *hookeri* and *Polyrhachis lydiae*	Australia
*Ophiocordyceps nooreniae*	BRIP 64868	KX961142	–	KX961143	–	**–**	*Polyrhachis* cf. *hookeri* and *Polyrhachis lydiae*	Australia
*Ophiocordyceps nutans*	OSC 110994	DQ522549	DQ518763	DQ522333	DQ522378	**–**	Hemiptera	–
***Ophiocordyceps nuozhaduensis***	**YHH 20168**	**ON555849**	**ON555927**	**ON567769**	**ON568683**	**–**	***Camponotus* sp.**	**China**
***Ophiocordyceps nuozhaduensis***	**YHH 20169**	**ON555850**	**ON555928**	**ON567770**	**ON568684**	**–**	***Camponotus* sp.**	**China**
*Ophiocordyceps odonatae*	TNSF 18563	**–**	KJ878877	**–**	KJ878992	**–**	Odonata	Japan
*Ophiocordyceps odonatae*	TNS 27117	**–**	KJ878878	**–**	**–**	**–**	Odonata	Japan
*Ophiocordyceps oecophyllae*	OECO1	KX713635	**–**	**–**	**–**	**–**	*Oecophyllas maragdina*	Australia
*Ophiocordyceps ootakii*	J14	KX713651	**–**	KX713682	KX713709	**–**	*Polyrhachis moesta*	Japan
*Ophiocordyceps ootakii*	J13	KX713652	KX713600	KX713681	KX713708	**–**	*Polyrhachis moesta*	Japan
*Ophiocordyceps ponerinarum*	HUA 186140^T^	KC610789	KC610767	KC610740	KF658668	**–**	*Paraponera clavata*	Brazil
*Ophiocordyceps pulvinata*	TNS-F 30044^T^	GU904208	**–**	GU904209	GU904210	**–**	*Camponotus obscuripes*	Japan
*Ophiocordyceps purpureostromata*	TNSF 18430	KJ878931	KJ878897	KJ878977	KJ879011	**–**	Coleoptera	Japan
*Ophiocordyceps polyrhachis-furcata*	P39	KJ201504	**–**	JN819003	**–**	**–**	*Polyrhachis furcata*	Thailand
*Ophiocordyceps polyrhachis-furcata*	P51	KJ201505	**–**	JN819000	**–**	**–**	*Polyrhachis furcata*	Thailand
*Ophiocordyceps ravenelii*	OSC 151914	KJ878932	**–**	KJ878978	KJ879012	KJ878950	Coleoptera	USA
*Ophiocordyceps rhizoidea*	NHJ 12529	EF468969	EF468824	EF468765	EF468872	EF468922	Coleoptera	–
*Ophiocordyceps rhizoidea*	NHJ 12522	EF468970	EF468825	EF468764	EF468873	EF468923	Coleoptera	–
*Ophiocordyceps rami*	MY6736^T^	KM655823	–	KJ201532	–	–	*Camponotus* sp.	Thailand
*Ophiocordyceps rami*	MY6738	KM655824	–	KJ201534	–	–	*Camponotus* sp.	Thailand
*Ophiocordyceps satoi*	J19	KX713650	KX713601	KX713684	KX713710	–	*Polyrhachis lamellidens*	Japan
*Ophiocordyceps satoi*	J7	KX713653	KX713599	KX713683	KX713711	–	*Polyrhachis lamellidens*	Japan
*Ophiocordyceps septa*	Pur1	–	–	KJ201528	–	–	*Camponotus* sp.	Thailand
*Ophiocordyceps septa*	Pur2	–	–	KJ201529	–	–	*Camponotus* sp.	Thailand
*Ophiocordyceps septa*	C41	–	–	JN819037	–	–	*Camponotus* sp.	Thailand
*Ophiocordyceps sinensis*	EFCC 7287	EF468971	EF468827	EF468767	EF468874	EF468924	Lepidoptera	–
*Ophiocordyceps sobolifera*	KEW 78842	EF468972	EF468828	–	EF468875	EF468925	Hemiptera	–
*Ophiocordyceps sphecocephala*	OSC 110998	DQ522551	DQ518765	DQ522336	DQ522381	DQ522432	Hymenoptera	–
*Ophiocordyceps stylophora*	OSC 111000	DQ522552	DQ518766	DQ522337	DQ522382	DQ522433	Coleoptera	–
*Ophiocordyceps stylophora*	OSC 110999	EF468982	EF468837	EF468777	EF468882	EF468931	Coleoptera	–
***Ophiocordyceps subtiliphialida***	**YFCC 8815**^T^	**ON555833**	**ON555914**	**ON567753**	**ON568673**	**ON568126**	***Camponotus* sp.**	**China**
***Ophiocordyceps subtiliphialida***	**YFCC 8814**	**ON555834**	**ON555915**	**ON567754**	**ON568674**	**ON568127**	***Camponotus* sp.**	**China**
***Ophiocordyceps subtiliphialida***	**YFCC 8816**	**ON555835**	**ON555916**	**ON567755**	**ON568675**	**ON568128**	***Camponotus* sp.**	**China**
***Ophiocordyceps subtiliphialida***	**YFCC 8817**	**ON555836**	**ON555917**	**ON567756**	**ON568676**	**ON568129**	***Camponotus* sp.**	**China**
*Ophiocordyceps tricentri*	CEM 160	AB027330	AB027376	–	–	–	Hemiptera	–
*Ophiocordyceps tianshanensis*	MFLU 19-1207^T^	MN025409	MN025407	MK992784	–	–	*Camponotus japonicu*s	China
*Ophiocordyceps tianshanensis*	MFLU 19-1208	MN025410	MN025408	MK992785	–	–	*Camponotus japonicus*	China
*Ophiocordyceps unilateralis*	VIC 44303	KX713628	KX713626	KX713675	KX713730	–	*Camponotus sericeiventris*	Brazil
*Ophiocordyceps unilateralis*	VIC 44354	KX713627	–	KX713676	KX713731	–	*Camponotus sericeiventris*	Brazil
*Ophiocordyceps yakusimensis*	HMAS_199604	KJ878938	KJ878902	–	KJ879018	KJ878953	Hemiptera	China
*Paraisaria amazonica*	HUA 186113	KJ917566	–	–	KP212903	KM411980	Orthoptera	Colombia
*Paraisaria gracilis*	EFCC 8572	EF468956	EF468811	EF468751	EF468859	EF468912	Lepidoptera	–
*Paraisaria gracilis*	EFCC 3101	EF468955	EF468810	EF468750	EF468858	EF468913	Lepidoptera	–
*Paraisaria heteropoda*	OSC 106404	AY489690	AY489722	AY489617	AY489651	–	Hemiptera	Australia
*Tolypocladium inflatum*	OSC 71235	EF469124	EF469077	EF469061	EF469090	EF469108	Coleoptera	–
*Tolypocladium ophioglossoides*	CBS 100239	KJ878910	KJ878874	KJ878958	KJ878990	KJ878944	*Elaphomyces* sp.	–

^T^Type material. New species were shown in bold

#### Phylogenetic analyses of ants

Phylogenetic analyses were based on *COI* gene sequences. Sequences of *COI* gene from various species (see Table 2) were retrieved from GenBank and the nucleotide sequences were combined with those generated in our study. Information on specimens and GenBank accession numbers were listed in Table 2. Sequences were aligned using Clustal X (v.2.0) (Larkin et al. 2007), poorly-aligned regions were removed and adjusted manually using MEGA6 (v.6.0) (Tamura et al. 2013). One host dataset (*COI*) was generated. Modelfinder (Kalyaanamoorthy et al. 2017) was used to select the best-fitting likelihood model for maximum likelihood (ML) analyses, and Bayesian inference (BI) analyses were carried out for the host datasets. The Corrected Akaike Information Criterion (AIC) was used to select the model for each gene, and the best-fitting models were provided in Table 3. The latter method was consistent with the phylogenetic analyses of fungi.

**Table 2:** The *COI* genes and GenBank accession numbers of the taxa were used in this study

Species name	Voucher information	GenBank number
*Camponotus americanus*	YNH-005	MZ331828
*Camponotus americanus*	BKH-019	MW802204
*Camponotus badia*	TUCIM:6601	MF993268
*Camponotus badia*	TUCIM:6461	MF993266
*Camponotus castaneus*	BIOUG03675-H07	KJ208900
*Camponotus castaneus*	BIOUG03675-H04	KJ445248
*Camponotus claripes*	AECT	JN134855
*Camponotus cylindricus*	–	EF634204
*Camponotus explodens*	TUCIM:5080	MF993254
*Camponotus novogranadensis*	–	MT904506
*Camponotus renggeri*	Creng_1_B	KP101600
*Camponotus rufipes*	BIOUG24424-D11	OM314604
*Camponotus saundersi*	–	BK012313
*Camponotus saundersi*	–	MT904541
*Camponotus simulans*	AFR-CND-2010-47-F02	JN270684
*Camponotus* sp*.*	CASENT0441197-D01	GU710187
*Camponotus* sp.	CASENT0043700-D01	KF200199
*Camponotus* sp.	CAMPO014	MH290634
*Camponotus* sp.	CASENT0000633-D01	HM373060
***Camponotus* sp.**	**YHH 20122**	**OP353539**
***Camponotus* sp.**	**YHH 20605**	**OP353540**
***Camponotus* sp*.***	**YHH 20606**	**OP353541**
***Camponotus* sp.**	**YHH 20607**	**OP353542**
***Camponotus* sp.**	**YHH 20608**	**OP353543**
***Camponotus* sp.**	**YHH 20609**	**OP353544**
***Camponotus* sp.**	**YHH 20610**	**OP353545**
***Camponotus* sp.**	**YHH 20611**	**OP353546**
***Camponotus* sp.**	**YHH 20612**	**OP353547**
***Camponotus* sp.**	**YHH 20168**	**OP353548**
***Camponotus* sp.**	**YHH 20191**	**OP353549**
*Camponotus spanis*	G191388	OM420293
*Camponotus sericeiventris*	BIOUG13980-G06	OM558348
*Camponotus sericeiventris*	BIOUG24738-E05	OM556713
*Camponotus sexguttatus*	CASENT0612243	JF863527
*Camponotus vitreus*	gvc13410-1L	HM914891
*Camponotus vitreus*	gvc13412-1L	HM914893
*Camponotus wiederkehri*	AEKB	JN134865
*Dolichoderus bispinosus*	–	KU187256
*Dolichoderus quadridenticulatus*	–	KU187255
*Dolichoderus bispinosus*	MACN-bar-ins-07510	MN625067
*Daceton armigerum*	USNM:ENT:01566820	MW983875
*Oecophylla smaragdina*	CSM0633	KM348012
*Oecophylla smaragdina*	EM898	MN619431
*Polyrhachis anderseni*	ANA42	KM348248
*Polyrhachis ammon*	RA0751	KY939110
*Polyrhachis aurea*	RA0750	KM348211
*Polyrhachis arnoldi isolate*	NDA40	MK591916
*Polyrhachis beccari*	FMNH-INS_2842133	KM348266
*Polyrhachis carbonaria*	FMNH-INS_2842101	KM348267
*Polyrhachis cf. bismarckensis*	FMNH-INS_2842022	KM348331
*Paraponera clavata*	YB-BCI150685	MK769309
*Polyrhachis cupreata*	CSM1015	KY939064
*Polyrhachis cupreata*	CSM0682	KY939056
*Polyrhachis flavibasis*	RA0766	KM348203
*Polyrhachis flavibasis*	RA0763	KY939081
*Polyrhachis furcata*	YB-KHC51412	MN618329
*Polyrhachis gagates*	FMNH-INS_2842213	KM348270
*Polyrhachis hookeri*	RA0747	KM348215
*Polyrhachis illaudata*	FMNH-INS_2842112	KM348275
*Polyrhachis illaudata*	FMNH-INS_2842222	KM348271
*Polyrhachis jianghuaensis*	GXBL0006	JQ681069
*Polyrhachis latharis*	FMNH-INS_2842062	KM348278
*Polyrhachis lamellidens*	NSMK-IN-170100347	OL663445
*Polyrhachis lucidula*	G160084	OM420302
*Polyrhachis mucronata*	RA1154	KM348338
*Polyrhachis mucronata*	RA1158	KM348339
*Polyrhachis mucronata*	RA1164	KM348340
*Polyrhachis mucronata*	CSM0696a	KM348337
*Polyrhachis nigropilosa*	FMNH-INS_2842045	KM348284
*Polyrhachis noesaensis*	FMNH-INS_2842106	KM348285
*Polyrhachis obesior*	FMNH-INS_2842054	KM348286
*Polyrhachis ornata*	CSM0797	KM348255
*Polyrhachis ornata*	CSM0842	KY939061
*Polyrhachis proxima*	G191229	OM420306
*Polyrhachis proxima*	FMNH-INS_2842042	KM348289
*Polyrhachis proxima*	FMNH-INS_2842129	KM348288
*Polyrhachis schistacea*	FMNH-INS_2842059	KM348296
*Polyrhachis schistacea*	FMNH-INS_2842058	KM348297
*Polyrhachis schistacea*	FMNH-INS_2842065	KM348295
*Polyrhachis schistacea*	FMNH-INS_2842071	KM348294
*Polyrhachis schistacea*	FMNH-INS_2842072	KM348292
*Polyrhachis schlueteri*	CASENT	KM348298
*Polyrhachis* sp.	RA0784	KM348355
*Polyrhachis* sp.	FMNH-INS_2842139	KM348305
*Polyrhachis* sp.	FMNH-INS_2842198	KM348309
*Polyrhachis* sp.	FMNH-INS_2842195	KM348308
*Polyrhachis* sp.	FMNH-INS_2842179	KM348300
*Polyrhachis* sp.	FMNH-INS_2842190	KM348304
*Polyrhachis* sp.	FMNH-INS_2842193	KM348310
*Polyrhachis* sp.	FMNH-INS_2842194	KM348307
*Polyrhachis* sp.	FMNH-INS_2842074	KM348226
*Polyrhachis* sp.	RA736b	KM348229
***Polyrhachis* sp.**	**YHH 20162**	**OP353532**
***Polyrhachis* sp.**	**YHH 20163**	**OP353533**
***Polyrhachis* sp.**	**YHH 20164**	**OP353534**
***Polyrhachis* sp.**	**YHH 20601**	**OP353535**
***Polyrhachis* sp.**	**YHH 20602**	**OP353536**
***Polyrhachis* sp.**	**YHH 20603**	**OP353537**
***Polyrhachis* sp.**	**YHH 20604**	**OP353538**
*Polyrhachis turneri*	CSM0722	KY939058
*Polyrhachis villipes*	FMNH-INS_28421186	KM348316

Boldface: data generated in this study

**Table 3:** Results of the best-fitting likelihood model for maximum likelihood (ML) and Bayesian inference (BI) for the two datasets

Gene name	ML	BI
SSU	TNe + I + G4	K2P + I + G4
LSU	GTR + F + I + G4	GTR + F + I + G4
*TEF*	GTR + F + I + G4	GTR + F + I + G4
*RPB1*	GTR + F + I + G4	GTR + F + I + G4
*RPB2*	TIM + F + I + G4	GTR + F + I + G4
*COI*	GTR + F + I + G4	GTR + F + I + G4

## RESULTS

### Phylogenetic analysis of the genus *Ophiocordyceps*

**Fig. 1:**
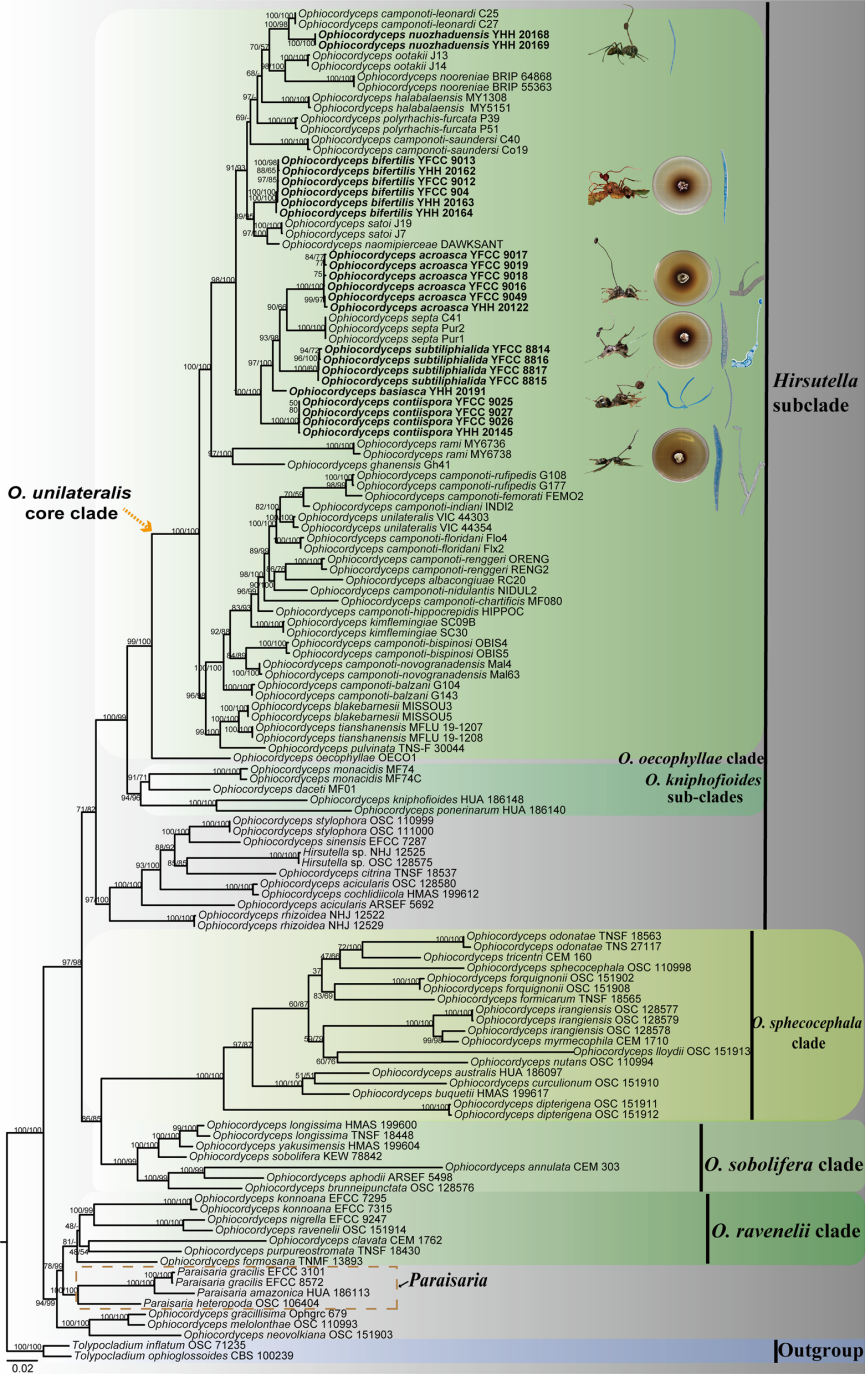
The phylogenetic tree of *Ophiocordyceps* and its related genera was inferred from five-gene dataset (*SSU*, *LSU*, *TEF*, *RPB1*, *RPB2*) based on Bayesian inference and maximum likelihood analyses. The illustration indicated to characteristics of new species. *Tolypocladium inflatum* OSC 71235 and *Tolypocladium ophioglossoides* CBS 100239 were designated as outgroups

Sequences of 129 samples were used for phylogenetic analysis. *Tolypocladium inflatum* OSC 71235 and *Tolypocladium ophioglossoides* CBS 100239 were designated as outgroups. The total length of the concatenated dataset of five genes across the 129 samples was 4785 bp, including 1057 bp for SSU, 952 bp for LSU, 965 bp for *TEF*, 738 bp for *RPB1*, and 1073 bp for *RPB2*. The phylogenetic relationships showed four clades in *Ophiocordyceps*, including the *Hirsutella* clade, *O. sphecocephala* clade, *O. sobolifera* clade and *O. ravenelii* clade. *Ophiocordyceps unilateralis* clade (34 species; BP = 100%, PP = 99%), *O. kniphofioides* sub-clade (3 species; BP = 94%, PP = 96%) and *O. oecophyllae* clade (1 species; BP = 99%, PP = 100%) were strongly supported by BI and ML analyses (Fig. 1). All the species collected and described in this work were clustered in the *O. unilateralis* core clade and clustered into a clade with *O. unilateralis *sensu lato species reported in Asian African (Ghana, Japan, Thailand) and Oceania (Australia) countries.

### Phylogenetic analysis of host ants

**Fig. 2:**
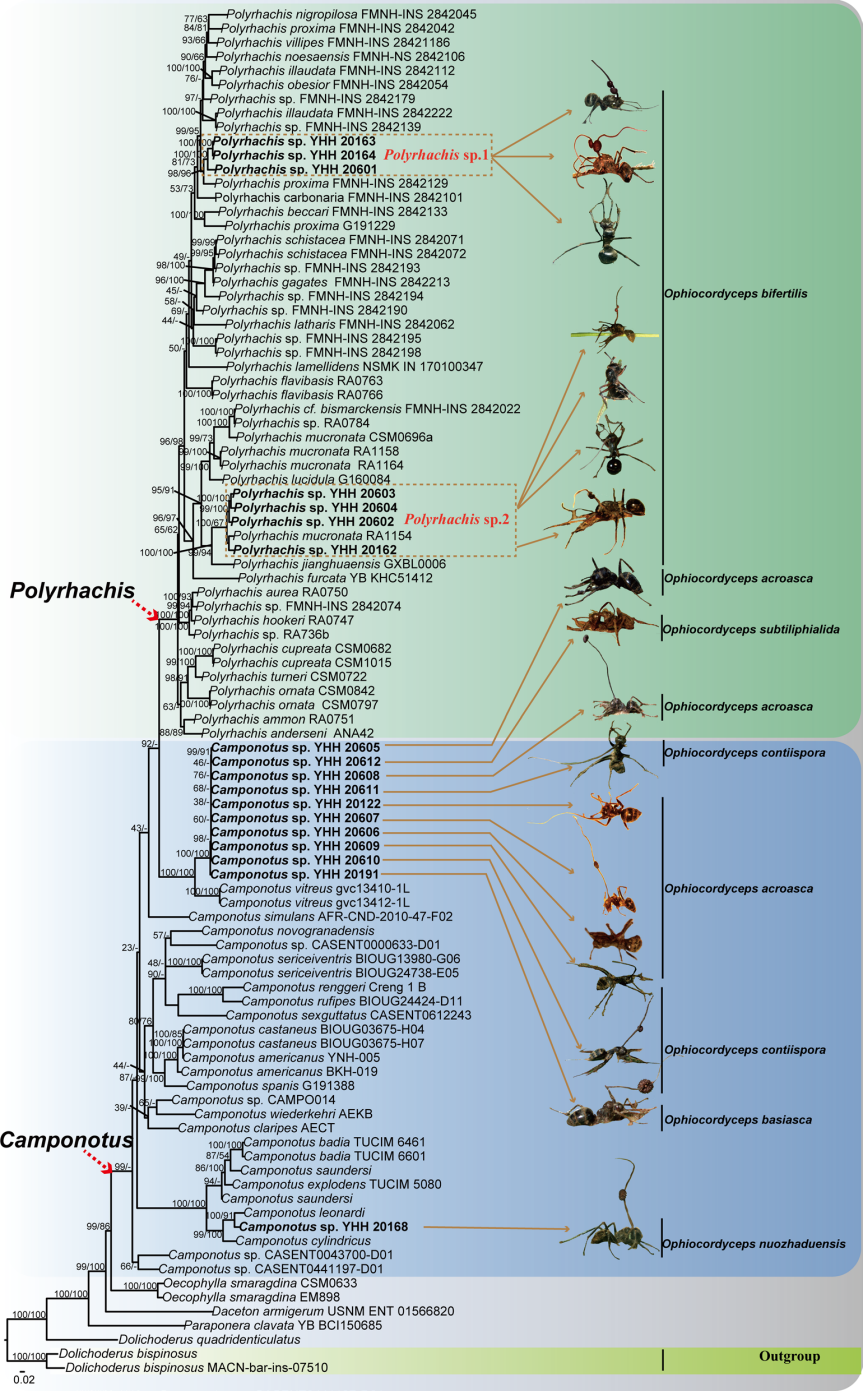
The phylogenetic tree of *Polyrhachis *and *Camponotus* including 97 taxa reconstructed using Bayesian inference and maximum likelihood. Each value at a node indicates a Bayesian posterior probability and bootstrap proportions. The Latin name refered to the pathogenic fungus that infected the host ant

Sequences of 97 specimens were used for phylogenetic analysis. *Dolichoderus bispinosus* was designated as the outgroup. Phylogenetic relationships have demonstrated that the phylogenetic trees consist of *Camponotus*, *Polyrhachis*, *Paraponera*, *Oecophylla* and *Dolichoderus*. Phylogenetic tree showed that *O. bifertilis* had two ant hosts (Fig. 2), namely, *Polyrhachis* sp.1 (*Polyrhachis* sp. YHH 20163, *Polyrhachis* sp. YHH 20164, *Polyrhachis* sp. 20601) and *Polyrhachis* sp.2 (*Polyrhachis* sp. YHH 20603, *Polyrhachis* sp. YHH 20604, *Polyrhachis* sp. YHH 20602, *Polyrhachis* sp. YHH 20162), with being a higher bootstrap value and posterior probability. *Camponotus leonardi* was sister to *Camponotus* sp. based on the host phylogenetic relationships*.* Their pathogenic fungi, such as *O. nuozhaduensis* and *O. camponoti-leonardi*, were also sister species. Notably, the phylogenetic relationships also showed that these ant pathogenic fungi, i.e., *O. basiasca*, *O. contiispora*, *O. acroasca*, *O. subtiliphialida*, parasitized on the same host *Camponotus* sp. (Fig. 2).

## TAXONOMY

### *Ophiocordyceps acroasca* Hong Yu bis & D.X. Tang, sp. nov.

**Fig. 3:**
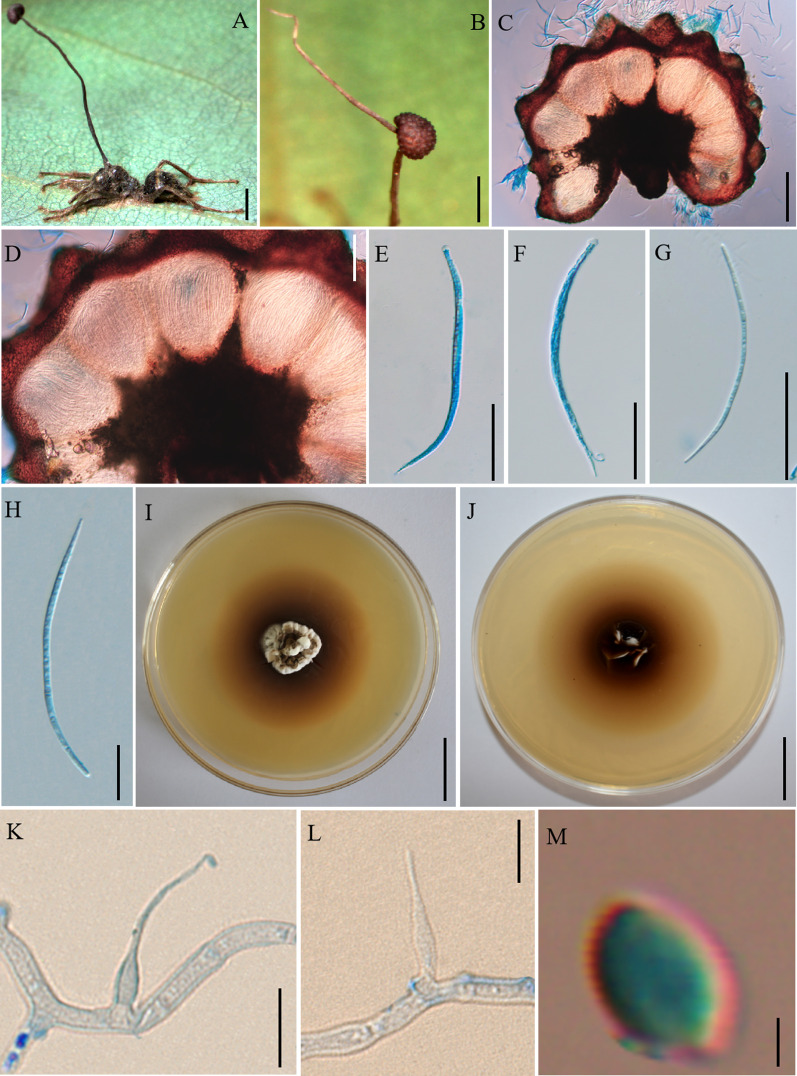
*Ophiocordyceps acroasca*. **A**: Infected *Camponotus* sp. was biting into a leaf of tree seedling. **B**: The ascoma was produced from the stroma. **C**, **D**: Cross-section of the ascoma showing the perithecial arrangement. **E**, **F**: Asci. **G**,** H**: Ascospores. **I**, **J**: Colonies on PDA medium. **K**, **L**: Conidiogenous cells and conidia. **M**: Conidia. Scale bars: **A** = 3000 µm; **B** = 2000 µm; **C** = 200 µm; **D** = 100 µm; **E–G** = 50 µm; **H** = 20 µm; **I**, **J** = 2 µm; **K**, **L** = 10 µm; **M** = 2 µm

Mycobank: MB 844350 (Fig. 3)

*Etymology*: The epithet refered to ascomata of lateral cushions produced from the top of stromata.

*Diagnosis*: Similar to *O. septa* in immersed and ostiole perithecia, but *O. acroasca* differs by ascomata arising from the top of stromata.

*Type*: **China:** Yunnan, Puer City, Sun River Natioal Park. *Camponotus* sp. was infected and bited into a leaf of tree seedling, 22°35′38″ N, 101°6′36″ E, alt. 1452 m, 18 Aug. 2020, Hong Yu bis (YHH 20121 – holotype preserved in the Yunnan Herbal Herbarium; living culture YFCC 9016 – ex-holotype stored in Yunnan Fungal Culture Collection).

*Description*: **Sexual morph:** External mycelia produced from the legs and body of the host. Stromata single and curved at the top, produced from dorsal pronotum of the ant, cylindrical, clavate, dark brown at maturity, the top was lighter than other parts of stromata. Fertile regions (ascomata) of lateral cushions produced from the top of stromata, one to two ascomata were found, hemispherical, brown, averaging 3 × 2–3 mm. Perithecia ovoid, immersed to partially erumpent, with short, exposed neck or rounded ostiole, 247–296 × (170–) 176–225 (–238) μm. Asci cylindrical, hyaline, curved, thick, 8-spored, (126–) 131–172 (–180) × 5–8 μm. Ascus caps hemispherical, prominent and small, 3–5 µm high and 4–6 µm wide. Ascospores vermiform, thin-walled, hyaline, 4–5-septate, slightly curved to sinuous, round to slightly tapered at the apex, (76–) 83–108 (–113) × 2–3 µm. **Asexual morph:** Colonies on PDA slow-growing, 26–27 mm diameter in 60 days at 25 °C, milky white to light brown, hard, with protuberant mycelial at the surface, the pigment produced around colonies, dark brown, reverse light brown to dark brown. Hyphae branched, septate, smooth-walled, hyaline. *Hirsutella* type-A and *Hirsutella* type-C produced from colonies, *Hirsutella* not examined from sutures and joints because the specimens were used to isolated strains. Conidiogenous cells monophialidic, produced from hyphae, smooth, swollen base, cylindrical to lageniform, tapering gradually or abruptly a long neck, slight bending, 17–30 × 1–4 µm. Conidia limoniform, solitary, hyaline, smooth-walled, 2–3 × 1–2 µm.

Germination process: No germination observed because the specimens were dried.

*Host*: *Camponotus* sp. (Formicinae)

*Habitat*: Subtropical monsoon evergreen broad-leaf forest. Infected *Camponotus* sp. was found biting into a leaf of tree seedling; from 0.5 to 2 m above the ground.

*Distribution*: China, Yunnan Province, Puer City

*Material examined*: **China:** Yunnan, Puer City, Sun River National Park. Infected ants were found biting into a leaf of tree seedling, 22°38′2″ N, 101°6′7″ E, alt. 1468 m, 19 Aug. 2020, Hong Yu bis (living culture YFCC 9017, YFCC 9018, YFCC 9019, YFCC 9049) and 22°34′34″ N, 101°6′24″ E, alt. 1095 m, 23 Aug. 2021, D.X. Tang (YHH 20122).

Notes: Phylogenetic analyses showed that *O. acroasca* formed a sister lineage with *O. septa*, and was clustered in the *O. unilateralis* core clade of *Hirsutella,* with strong statistical supported by bootstrap proportions (BP = 90%) (Fig. 1). *Ophiocordyceps acroasca* was similar to *O. septa* in the behavior of the host biting a leaf, cylindrical or clavate stromata, immersed and ostiole perithecia. However, it differed from *O. septa* by ascomata of lateral cushion arising from the top of stromata, vermiform ascospores, producing *Hirsutella* type-A and *Hirsutella* type-C, cylindrical to lageniform conidiogenous cells, limoniform conidia. In addition, the sizes of perithecia, ascomata, asci, ascospores, phialides, and conidia also differed from *O. septa* (Table 4).

**Table 4:** Comparison of morphological characters and host of *Ophiocordyceps unilateralis *sensu lato in this study

Species	Host	Death position	Stromata	Ascomata	Perithecia (μm)	Asci (μm)	Prominent caps	Ascospores (μm)	Septa	*Hirsutella* asexual morph (μm)	Conidia (μm)	Country	References
***Ophiocordyceps acroasca***	***Camponotus* sp.**	**Biting leaf**	**Single**	**Hemispherical, 3 × 2–3 mm**	**Ovoid, 247–296 × 176–225**	**Cylindrical, 8-spored, 131–172 × 5–8**	**Prominent, 3–5 × 4–6**	**Vermiform, 83–108 × 2–3**	**4–5**	***Hirsutella* A-type and *Hirsutella* C-type, 17–30 × 1–4**	**Limoniform, 2–3 × 1–2**	**China**	**This study**
*Ophiocordyceps albacongiuae*	*Camponotus* sp.	Biting epiphites	One or two	Disc-shaped	Flask-shaped, 240–290 × 105–135	Cylindrical to clavate, 8-spored, 130–160 × 8–11	Hemispherical, 3–5 × 4–5	Cylindrical, 80–100 × 5	5–6	–	–	Colombia	Araújo et al. (2018)
***Ophiocordyceps basiasca***	***Camponotus* sp.**	**Biting leaf**	**Single**	**Spherical, 3 × 2 mm**	**Flask-shaped or ovoid, 202–242 × 102–149**	**Cylindrical, 8-spored, 96–188 × 4–9**	**Hemispherical, 3–5 × 4–5**	**Vermiform, 89–119 × 2–3**	**4–5**	***Hirsutella* A-type, 10–23 × 1–5**	**Oviform, 1–4 × 1–2**	**China**	**This study**
***Ophiocordyceps bifertilis***	***Polyrhachis* sp.1 and *Polyrhachis* sp.2**	**Biting leaf**	**Multiple**	**Disc-shaped or hemispherical, 3 × 2–3 mm**	**Flask-shaped, 156–211 × 102–129**	**Cylindrical, 8-spored, 130–198 × 6–10**	**Prominent, 3–5 × 5–6**	**Fusiform, 70–94 × 2–4**	**4–5**	***Hirsutella* A-type, 9–24 × 2–4**	**–**	**China**	**This study**
*Ophiocordyceps blakebarnesii*	*Camponotus* sp*.*	Biting inside log	Single	Discshaped to irregular, 1.5 × 1 mm	Flask-shaped, 300–320 × 105–120	Cylindrical to clavate, 8-spored, 220–250 × 12–14	–	Cylindrical, 140–160 × 4	6–7	*Hirsutella* A-type, 75 × 3–4	Limoniform, 8–9 × 3	USA	Araújo et al. (2018)
***Ophiocordyceps contiispora***	***Camponotus* sp.**	**Biting leaf**	**Single**	**Disc-shaped, 0.7–1 mm**	**Flask-shaped, 158–212 × 69–122**	**Cylindrical, 8-spored, 89–130 × 4–9**	**Hemispherical or square, 1–3 × 3–5**	**Fusiform, 38–48 × 2–4**	**No obvious separation**	***Hirsutella* C-type, 57–92 × 1–4**	**Olivary or flask-shaped, 4–6 × 1–2**	**China**	**This study**
*Ophiocordyceps camponoti-leonardi*	*Camponotus leonardi*	Biting leaf	Single	–	Fusoid-ellipsoid, 400–430 × 200–230	Cylindrical, 8-spored, 130–175 × 7–8	–	Lanceolate, 110–125 × 2–3	Multiseptate	*Hirsutella*, 22.5 × 2.0–3.5	Fusoid 2–4 × 1–2	Thailand	Kobmoo et al. (2012)
*Ophiocordyceps camponoti-saundersi*	*Camponotus saundersi*	Biting leaf	Single	–	Fusoid-ellipsoid, 280–320 × 160–180	Cylindrical, 8-spored, 80–160 × 6–7	–	Lanceolate, 75–85 × 2–3	Multiseptate	*Hirsutella*, 25 × 2–3	Fusoid, 2–3 × 1–2	Thailand	Kobmoo et al. (2012)
*Ophiocordyceps halabalaensis*	*Camponotus gigas*	Biting leaf	Three	–	Fusoid-ellipsoid, 350–420 × 180–210	Cylindrical, 8-spored, 150–200 × 7–10	–	Cylindrical, 60–75 × 3–5	Multiseptate	–	–	Thailand	Luangsa-ard et al. (2011)
*Ophiocordyceps nooreniae*	*Polyrhachis cf. hookeri*	Biting leaf	–	–	–	–	–	–		*Hirsutella* A-type, 30–55; *Hirsutella* C-type, 35–50 × 1.5–8	Ovoid, 5–6 × 2–3	Australia	Crous et al. (2016)
***Ophiocordyceps nuozhaduensis***	***Camponotus* sp.**	**Biting leaf**	**Single**	**Spherical, 2.4 × 1.6 mm**	**Flask-shaped, 222–274 × 153–159**	**–**		**Vermiform, 91–126 × 2–5**	**7–13**	***Hirsutella* A-type, 6–22 × 2–4**	**Ellipsoidal or oviform, 2–5 × 2–3**	**China**	**This study**
*Ophiocordyceps naomipierceae*	*Polyrhachis cf. robsonii*	Biting leaf	–	Hemispherical to irregular, 0.75 × 0.5–0.65 mm	Flask-shaped, 260–320 × 150–200	Vermiform, cylindrical, 8-spored, 150–180 × 7	Prominent	Vermiform, 75–105 × 5–6	4–6	*Paraisaria*-like, 15–35 × 3	Conidium, 5–7 × 3	Australia	Araújo et al. (2018)
*Ophiocordyceps ootakii*	*Polyrhachis* sp.	Biting leaf	Single or branched	Fisc-shaped, 1.1 × 0.8 mm	Flask-shaped, 230–260 × 120–150	Cylindrical to clavate, 8-spored, 130–180 × 8–9	Prominent	Vermiform, 85–100 × 3	5	*Hirsutella* A-type, 6–8 × 3–4	5 × 3	Japan	Araújo et al. (2018)
*Ophiocordyceps polyrhachis-furca*	*Polyrhachis furca*	Biting leaf	Single	–	Fusoid-ellipsoid, 380–400 × 160–180	Cylindrical, 8-spored, 140–190 × 7–8 µm	–	Lanceolate, 90–100 × 2–3	Multi-septate	*Hirsutella*, 30 × 2–3	Fusoid, 3–5 × 2–3	Thailand	Kobmoo et al. (2012)
*Ophiocordyceps rami*	*Camponotus* sp.	Biting leaf	Single	Hemispherical, 2 mm	Fusoid-ellipsoid, 325–500 × 275–300	Cylindrical, 8-spored, 200–340 × 7–10	–	Filiform, 200–215 × 2–3	7–8	*Hisutella* A-type, 9–10 × 3–4; *Hisutella* C-type, 30 × 3–5	Cylindrical to narrow fusiform, 3.5–6.5 × 1–2; fusiform to narrowly lemoniform, 9 × 5	Thailand	Kobmoo et al. (2015)
*Ophiocordyceps satoi*	*Polyrhachis lamellidens*	Biting twing	Three	1 × 0.8 mm	Flask-shaped, 230–270 × 120–160	Cylindrical to clavate, 8-spored, 120–160 × 8–10	Prominent	Cylindrical, 85–100 × 4	5	*Hirsutella* A-type, 12 × 7	–	Japan	Araújo et al. (2018)
*Ophiocordyceps septa*	*Camponotus* sp.	Biting leaf	Single	Hemispherical, 2 mm	Fusoid-ellipsoid, 280–300 × 100–150	Cylindrical, 8-spored, 125–165 × 12.5–15	–	Lanceolate, 45–50 × 6–8	7–8	*Hisutella* A-type, 25 × 2–3; *Hisutella* C-type, 50 × 5.5	Fusiform, 5–6 × 1–2; fusiform to narrowly lemoniform, 9 × 5	Thailand	Kobmoo et al. (2015)
***Ophiocordyceps subtiliphialida***	***Camponotus* sp.**	**Biting leaf**	**Single**	**Disc-shaped, 2 × 1.2–1.9 mm**	**Flask-shaped, 195–296 × 87–161**	**Cylindrical, 8-spored, 89–119 × 5–9**	**Hemispherical, 2–4 × 5–7**	**Lanceolate, 52–72 × 5–8**	**6–7**	***Hirsutella* C-type, 70–116 × 1–3**	**Olivary, 6–10 × 3–6**	**China**	**This study**
*Ophiocordyceps camponoti-atricipis*	*Camponotus atriceps*	Biting leaf	Single	Hemispherical, 1.5 × 0.5–0.8 mm	Flask-shaped, 240–280 × 100–150	Cylindrical to clavate, 8-spored, hyaline, 110–140 × 6–6.5	5 × 5.5	Vermiform 80–85 × 3	5	*Hirsutella* A-type, 5–7 × 2–3	–	Brazil	Araújo et al. (2015)
*Ophiocordyceps camponoti-balzani*	*Camponotus balzani*	Biting leaf	Single	1.5 × 1.0 mm	Flask-shaped, 400–450 × 100–150	Cylindrical, 8-spored, 200–240 × 12–16	Prominent, 8–10 × 6–8	Cylindrical, 135–175 × 4.0–5.0	14–22	*Hirsutella* A-type, *Hirsutella* C-type 20–25 × 3–4	Cylindric to fusiform, 12–14 × 2–3	Brazil	Evans et al. (2011b)
*Ophiocordyceps camponoti-bispinosi*	*Camponotus bispinosus*	Biting spines	Single	0.8 × 0.4–0.7 mm	Globose to flask-shaped, 250–290 × 150–170	Cylindrical to clavate, 8-spored, hyaline, 110–130 × 8–8.5	3.5 × 4.5	Cylindrical, 70–75 × 4.5–5	4–5	*Hirsutella* A-type, 6 × 2.5–3	Narrow limoniform, 6–7 × 2	Brazil	Araújo et al. (2015)
*Ophiocordyceps camponoti-chartificis*	*Camponotus chartifex*	Biting leaf	Single	Hemispherical, 1.5 × 1 mm	Globose to hemispherical shaped, 200–235 × 135–175	Cylindrical to clavate, 8-spored, 100–125 × 6	6–7 × 3–4	Vermiform 75–85 × 5	9–13	*Hirsutella* A-type, 5–6 × 3	Fusiform to limoniform, 7 × 2.6	Brazil	Araújo et al. (2018)
*Ophiocordyceps camponoti-femorati*	*Camponotus femoratus*	Biting leaf/spines	Single	Disc-shaped to hemispherical, 1.2–2.2 × 0.8–1.4 mm	Flask-shaped, 200–230 × 135–165	Cylindrical to clavate, 8-spored, 110–130 × 8–9	6 × 3	75–90 × 3	5	*Hirsutella* A-type, 7–10 × 3–4	Limoniform, 7–9 × 3	Brazil	Araújo et al. (2018)
*Ophiocordyceps camponoti-floridani*	*Camponotus floridanus*	Biting leaf	Single	Disc-shaped	Flask-shaped, 265 × 100	Cylindrical to clavate, 8-spored, 145 × 9–10	–	Cylindrical, 75–90 × 4–5	5	*Hirsutella* A-type, 8–9 × 3–4	Limoniform, 8–9 × 3	USA	Araújo et al. (2018)
*Ophiocordyceps camponoti-hippocrepidis*	*Camponotus hippocrepis*	Biting spines	Single	2–2.5 × 0.25–0.45 mm	Flask-shaped, 225–250 × 135–165	Cylindrical to clavate, 8-spored, 115–135 × 7–10	Prominent, 6–7 × 4	Cylindrical, 75–85 × 4–5	5	*Hirsutella* A-type, 8–9 × 4	Limoniform, 5 × 2	Brazil	Araújo et al. (2018)
*Ophiocordyceps camponoti-indiani*	*Camponotus indianus*	Biting leaf	Multiple	Hemispherical	Ovoid to flask-shaped, 230–310 × 120–175	Cylindrical, 8-spored, 170 × 8.5	Prominent, 4.5 × 5	Cylindrical, 75 × 4.5	5	*Hirsutella* A-type, 7.5 × 3.5; *Hirsutella* C-type	–	Brazil	Araújo et al. (2015)
*Ophiocordyceps camponoti-melanotici*	*Camponotus melanoticus*	Biting leaf	Single	1.3 × 0.8 mm	Flask-shaped, 400–450 × 100–150	8-spored, 200–275 × 12–16	8–10 × 6–8	Cylindrical, 170–210 × 4–5	27–35	*Hirsutella* A-type	–	Brazil	Evans et al. (2011b)
*Ophiocordyceps camponoti-nidulantis*	*Camponotus niduland*	Biting saplings	Single	Disc-shaped to hemispherical, 1.5 × 1 mm	Flask-shaped, 200–240 × 100–150	Vermiform to clavate, 8-spored, 110–145 × 6–8	4 × 6	Vermiform, 90–105 × 3–4	5	*Hirsutella* A-type; *Hirsutella* C-type, 70–120 × 4–6	Limoniform, 8 × 3	Brazil	Araújo et al. (2015)
*Ophiocordyceps camponoti-novogranadensis*	*Camponotus novogranadensis*	Biting epiphites	Single	0.8–1.0 × 0.5–0.6 µm	225–250 × 125–155	Cylindrical, 8-spored, 95–120 × 9–10	Prominent, 5–6 × 3–4	Filiform, 75–95 × 2.5–3.5	5–10	*Hirsutella* A-type; *Hirsutella* B-type, 80–100 × 35–40	Narrowly clavate to obclavate, 10–12 × 1.5–2.0	Brazil	Evans et al. (2011b)
*Ophiocordyceps camponoti-renggeri*	*Camponotus renggeri*	Biting leaf/moss	Single	Hemispherical to globose, 1–1.5 × 0.8–1 mm	Flask-shaped, 220–250 × 100–165	Cylindrical, 8-spored, 130–145 × 8–10	Prominent, 7–8 × 3	Vermiform 90–120 × 4	5–8	*Hirsutella* C-type, 40–60 × 3–5	–	Brazil	Araújo et al. (2018)
*Ophiocordyceps camponoti-rufipedis*	*Camponotus rufipes*	Biting leaf	Single	Discshaped to hemisphaerical, 1 × 0.5 mm	Flask-shaped, 175–260 × 100–130	Cylindrical to clavate, 8-spored, 120–160 × 8–10	Prominent, 4.0–5.5 × 3.0–4.5	Vermiform, 80–95 × 2–3	4–7	*Hirsutella* A-type, 10 × 2	Fusiform to narrowly limoniform, 5 × 1.5	Brazil	Evans et al. (2011b)
*Ophiocordyceps camponoti-sexguttati*	*Camponotus sexguttatus*	Biting leaf	Single	Disc-shaped, 1 × 1 mm	Flask-shaped, 225–230 × 135	Cylindrical, 8-spored, 150–160 × 8–9	Prominent, 6 × 3	Cylindrical, 120–140 × 3	7	*Hirsutella* A-type, 5–8 × 3–4	Limoniform, 5 × 2	Brazil	Araújo et al. (2018)
*Ophiocordyceps kimflemingiae*	*Camponotus castaneus*	Biting twig	Single	Disc-shaped, 1.5–2 × 1.3 mm	Flask-shaped, 250–275 × 120–160	Cylindrical to clavate, 8-spored, 120–150 × 10–11	Prominent	Cylindrical, 80–90 × 5	5–6	*Hirsutella* A-type; *Hirsutella* C-type	–	USA	Araújo et al. (2018)
*Ophiocordyceps oecophyllae*	*Oecophylla smaragdina*	Biting leaf	–	–	–	–	–	–	–	30–50 × 3–4	Ovoid to cylindrical, 5.5–10 × 1.5–3	Australia	Araújo et al. (2018)
*Ophiocordyceps monacidis*	*Dolichoderus bispinosus*	Base of trunk	Single	–	–	–	–	–	–	–	–	Brazil	Araújo et al. (2018)
*Ophiocordyceps daceti*	*Daceton armigerum*	Leaf (not biting)	Single	–	–	–	–	–	–	*Hirsutella*, 16–18 × 4	Cylindrical, 7–10 × 3	Brazil	Araújo et al. (2018)
*Ophiocordyceps kniphofioides*	*Cephalotes atratus*	Base of trunk	Single	5–6 × 0.7–1 mm	Ovoid to lageniformia, 170–250 × 110–140	Narrow cylindrical, 140–200 × 6–12	–	Filiform, 110–150 × 1.5–3	3–5	*Hirsutella* A-type, 10–16 × 0.6–4; *Hirsutella* B-type	Narrowly clavate, 7–9 × 1.5–2.5; ovoid to cylindrical, 8–12 × 4–5	Brazil	Evans and Samson (1982)
*Ophiocordyceps ponerinarum*	*Paraponera clavata*	Base of trunk	–	8–14 × 0.8–1 mm	Flask-shaped, 210–320 × 140–190	–	–	–	–	*Hirsutella* A-type, 10–14 × 1.8–2.5	Clavate, 7–9 × 1.8–3	Brazil	Evans and Samson (1982)
*Ophiocordyceps pulvinata*	*Camponotus obscuripes*	Clinging to twigs	Single	–	400–600 × 150–250	Clavate, 8-spored, 220–300 × 9–19	4–5.4 × 6–9	Filiform, 160–220 × 3–5	–	–	–	Japan	Kepler et al. (2011)
*Ophiocordyceps tianshanensis*	*Camponotus japonicus*	The bark of a dilapidated (not biting)	–	Disc-shaped, 1.1–1.6 × 0.5–1.1 mm	Flask-shaped, 220–260 × 100–140	–	–	–	–	*Hirsutella* A-type, 8–9 × 2.5–3.5	Fusiform to obpyriform, 6–9.2 × 2.2–3	China	Wei et al. (2020)
*Ophiocordyceps unilateralis*	*Camponotus sericeiventris*	Biting leaf	Single	–	Flask-shaped, 200–250 × 140–160	Cylindrical, 8-spored, 95–125 × 6–8	5–6 × 4–5	Filiform, 75–85 × 2–2.5	4–5	*Hirsutella* A-type, 10–12 × 3–3.5; *Hirsutella* B-type, 14–16 × 2.5–3	Limoniform, 6.5–8 × 2–2.5; cylindrical-fusoid, 8–11 × 2.5–3	Brazil	Evans et al. (2018)

New species are shown in bold

### *Ophiocordyceps bifertilis* Hong Yu bis & D.X. Tang, sp. nov.

**Fig. 4:**
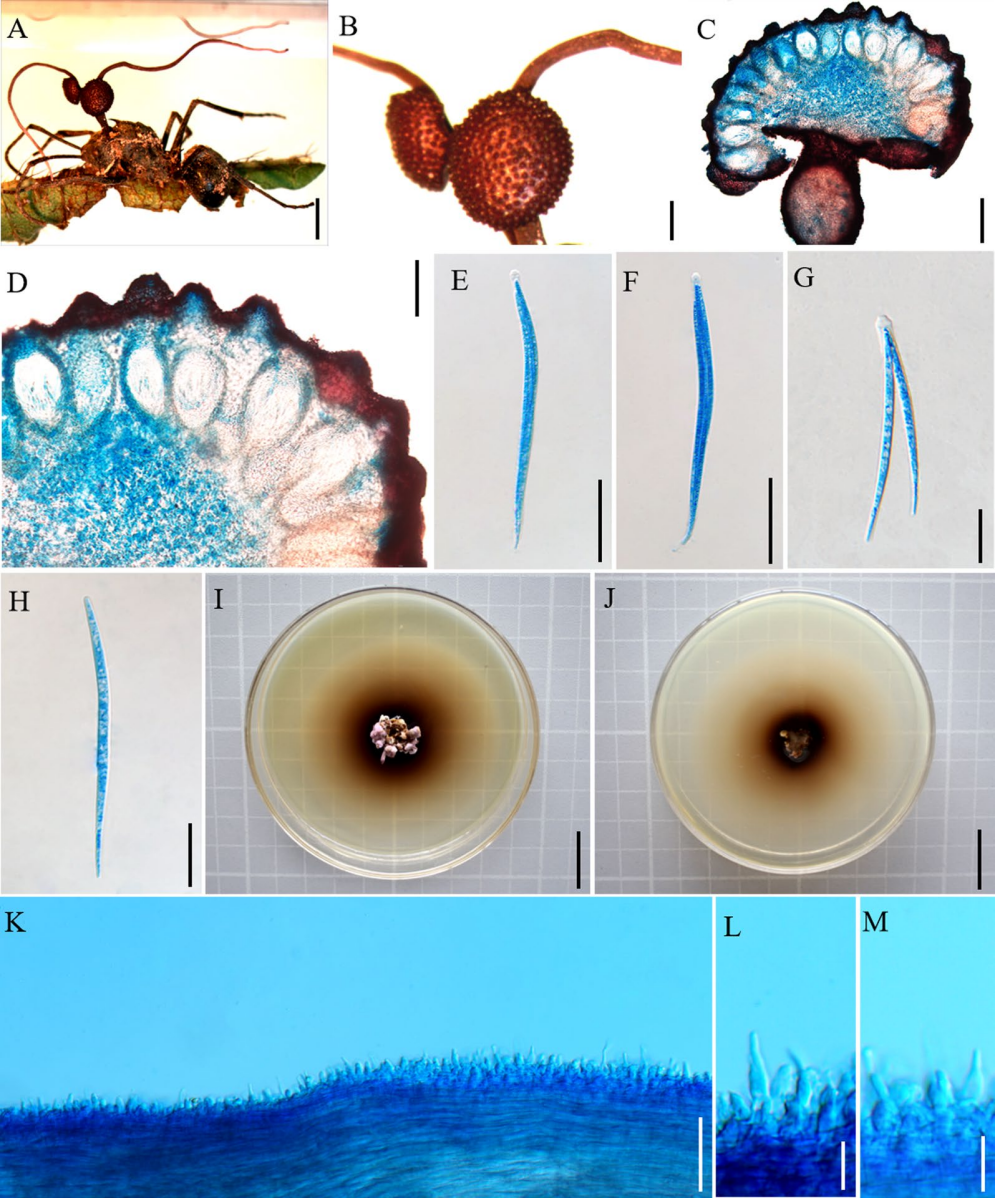
*Ophiocordyceps bifertilis*. **A**: Infected *Polyrhachis* sp.1was biting a leaf of Pteridophyta. **B**: Two ascomata plates attached to stromata. **C**, **D**: Cross-section of the ascoma showing the perithecial arrangement. **E**, **F**: Asci. **G**, **H**: Ascospores. **I**, **J**: Colonies on PDA medium. **K**, **L**: Phialides. Scale bars: **A** = 4000 µm; **B** = 1000 µm; **C** = 200 µm; **D** = 100 µm; **E**, **F** = 50 µm; **G**, **H** = 20 µm; **I**, **J** = 2 cm; **K–M** = 10 µm

Mycobank: MB 844351 (Fig. 4)

Etymology: The epithet refered to two fertile regions produced from stromata.

*Diagnosis*: *Ophiocordyceps bifertilis* similar to *O. satoi* regarding the production of multiple stalks, but *O. bifertilis* differed by stromata branching, with only two ascomata.

*Type*: **China:** Yunnan, Puer City, Sun River National Park. An adult *Polyrhachis* sp. was hanging upside down on the underside of the leaves, 2°20′24″ N, 101°6′43″ E, alt. 1487 m, 18 August 2020, Hong Yu bis (YHH 20160 – holotype preserved in the Yunnan Herbal Herbarium; living culture YFCC 9012 – ex-holotype stored in Yunnan Fungal Culture Collection).

*Description*: **Sexual morph:** External mycelia scarce, produced from sutures and joints. One to multiple stromata at the head of the ant, few branching, curved, cylindrical, clavate, dark brown. Ascomata of lateral cushions produced from stromata, two ascomata were observed, disc-shaped or hemispherical, brown, averaging 3 × 2–3 mm. Perithecia flask-shaped, immersed to partially erumpent, with short, exposed neck or rounded ostiole, (149–) 156–211 (–236) × (91–) 102–129 (–134) μm. Asci cylindrical, hyaline, 8-spored, (123–) 130–198 (–211) × 6–10 μm. Ascus caps were hemispherical, prominent, 3–5 µm high, and 5–6 µm wide. Ascospores fusiform, hyaline, 4–5-septate, round to tapered at the apex, 70–94 (–96) × 2–4 µm. **Asexual morph:** Colonies on PDA grows slowly, 19–20 mm diameter in 120 days at 25 °C, light purple to light brown, hard, with protuberant mycelia at the edge, reverse light brown to dark brown, pigment light brown to dark brown. *Hirsutella* type-A was present along stromata; *Hirsutella* was not observed from the sutures and joints. Phialides lageniform, smooth, swollen base, tapering abruptly a neck, short, 9–24 (–29) × 2–4 µm. Conidia were not observed.

Germination process: No germination observed because the specimens were dried.

*Host*: *Polyrhachis* sp.1 and *Polyrhachis* sp.2 (Formicinae)

*Habitat*: Subtropical monsoon evergreen broad-leaf forest. Infected *Polyrhachis* sp.1 was found biting into a leaf of Pteridophyta, and *Polyrhachis* sp.2 biting into a leaf of Gramineae, always at lower heights, ranging from 0.5 to 1.5 m.

*Distribution*: China, Yunnan Province, Puer City

*Material examined*: **China:** Yunnan, Puer City, Sun River National Park. Adult *Polyrhachis* sp.1 and *Polyrhachis* sp.2 were hanging upside down on the underside of the leaves of Pteridophyta and Gramineae, 22°35′50″ N, 101°6′39″ E, alt. 1529 m, 19 Aug. 2020, Hong Yu bis (living culture YFCC 9013, YFCC 9048) and 22°35′51″ N, 101°6′40″ E, alt. 1532 m, 23 Aug. 2021, D.X. Tang (YHH 20162, YHH 20163, YHH 20164).

Notes: Phylogenetic analyses revealed that *O. bifertilis* formed a sister lineage with *O. satoi* and *O. naomipierceae*, was clustered in the *O. unilateralis* core clade of *Hirsutella*, with statistical support from BI posterior probabilities (PP = 95%) and ML bootstrap proportions (BP = 89%) (Fig. 1). *Ophiocordyceps bifertilis* was similar to *O. satoi* and *O. naomipierceae* in the behavior of the host *Polyrhachis* infected and biting a leaf. In addition, it was also similar to *O. satoi* in clavate stromata, flask-shaped perithecia, *Hirsutella* type-A, lageniform phialides. However, it differed from *O. satoi* by branching stromata, fusiform ascospores. Moreover, the sizes of phialides also differed from *O. satoi* and *O. naomipierceae* (Table 4).

### *Ophiocordyceps subtiliphialida* Hong Yu bis & D.X. Tang, sp. nov.

**Fig. 5:**
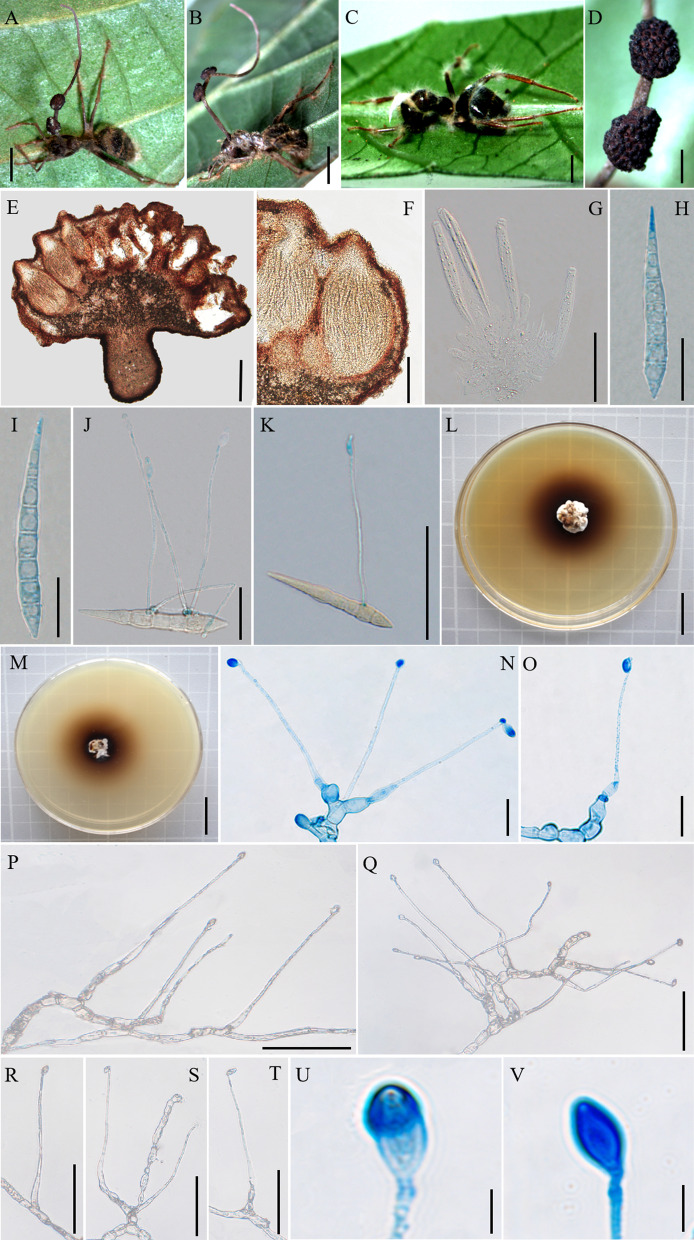
*Ophiocordyceps subtiliphialida*. **A**, **C**: *Camponotus* sp. was infected and bited into a leaf of a sapling. **D**: Fertile structure produced from the stroma; **E–F:** Cross-section of the ascoma showing the perithecial arrangement; **G**: Asci; **H**, **I**: Ascospores. **J**, **K**: Ascospore with long capilliconidia. **L**, **M**: Colonies on PDA medium. **N**–**V**: Conidiogenous cells and conidia. Scale bars: **A**, **B** = 0.4 cm; **C** = 0.2 cm; **D** = 0.1 cm; **E** = 200 µm; **F** = 100 µm; **G** = 50 µm; **H**–**J** = 20 µm; **K** = 50 µm; **L**, **M** = 2 cm; **N**, **O** = 20 µm; **P**–**T** = 50 µm; **U**, **V** = 5 µm

Mycobank: MB 844352 (Fig. 5)

Etymology: The epithet refered to the phialides slender than related species.

*Diagnosis*: Similar to *O. contiispora* in phialides monophialidic or rarely polyphialidic, but phialides of *O. subtiliphialida* (70–116 × 1–3 µm) was slender than *O. contiispora* (57–92 × 1–4 µm).

*Type*: **China:** Yunnan, Puer City, Sun River National Park. *Camponotus* sp. was infected and bited into a leaf of tree seedling, 22°34′34″ N, 101°6′24″ E, alt. 1420 m, 18 Aug. 2020, Hong Yu bis (YHH 20139 – holotype preserved in the Yunnan Herbal Herbarium; living culture YFCC 8815 – ex-holotype stored in Yunnan Fungal Culture Collection).

*Description*: **Sexual morph:** External mycelia produced from the sutures and joints of the ant. Stromata single, produced from dorsal pronotum of the ant, cylindrical, clavate, brown at maturity. Fertile part of lateral cushions produced from stromata, 1–2, disc-shaped, brown, averaging 2 × 1.2–1.9 mm. Perithecia flask-shaped, immersed to partially erumpent, with short, exposed ostiole, (195–) 199–296 (–303) × (87–) 97–161 (–168) μm. Asci cylindrical, hyaline, short and wide, 8-spored, 89–119 × 5–9 μm. Ascus caps hemispherical, 2–4 µm high, 5–7 µm wide. Ascospores lanceolate, hyaline, 6–7-septate, slightly curved, round to tapered at the apex, 52–72 × 5–7 (–8) µm. **Asexual morph:** Colonies grows slowly on PDA medium, 19–20 mm diameter in 60 days at 25 °C, milky white to light brown, raising cottony-shaped mycelia density at the edge, protuberant mycelia light brow at the centrum, reverse light brown to dark brown. Hyphae immersed in the medium, milky white, branched, septate, smooth-walled, hyaline. *Hirsutella* type-C only. Conidiophores rare, cylindrical, produced from the hyphae, septate, short and wide. Phialides monophialidic or rarely polyphialidic, forming on side hyphae or the conidiophores, smooth, slight swollen base, lageniform, septate, tapering gradually a slender neck, slight bending, 70–116 (–124) × 1–3 µm. Conidia olivary, solitary, hyaline, smooth-walled, 6–10 × 3–6 µm.

Germination process: Ascospores germinating in 72 h to produce 1–4, long and narrow capilliconidiophore, (44–) 58–79 μm long, 0.8–1.9 μm wide, bearing a single capilliconidium, averaging (6–) 7–9 × 2–3 μm.

*Host*: *Camponotus* sp. (Formicinae).

*Habitat*: Subtropical monsoon evergreen broad-leaf forest. Infected *Camponotus* sp. was found biting into a leaf of a sapling. Died in the lower position, collected from 0.5 to 1 m.

*Distribution*: China, Yunnan Province, Puer City.

*Material examined*: **China:** Yunnan, Puer City, Sun River National Park. ​Infected *Camponotus* sp. was found biting into a leaf of a sapling, 22°35′51″ N, 101°6′40″ E, alt. 1430 m, 19 Aug. 2020, Hong Yu bis (living culture YFCC 8814, YFCC 8816, YFCC 8817).

Notes: Phylogenetic analyses showed that the four samples of the *O. subtiliphialida* group together with high statistical support (PP = 60%; BP = 100%), were clustered within the *O. unilateralis* core clade of Southeast Asian countries (Fig. 1). It was similar to *O. septa*, *O. acroasca* and *O. basiasca* in swollen and lageniform base. However, it differed from *O. septa*, *O. acroasca* and *O. basiasca* by lanceolate ascospores, rare conidiophores, monophialidic or rarely polyphialidic phialides, tapering a narrow and slender neck, olivary conidia.

### *Ophiocordyceps basiasca* Hong Yu bis & D.X. Tang, sp. nov.

**Fig. 6:**
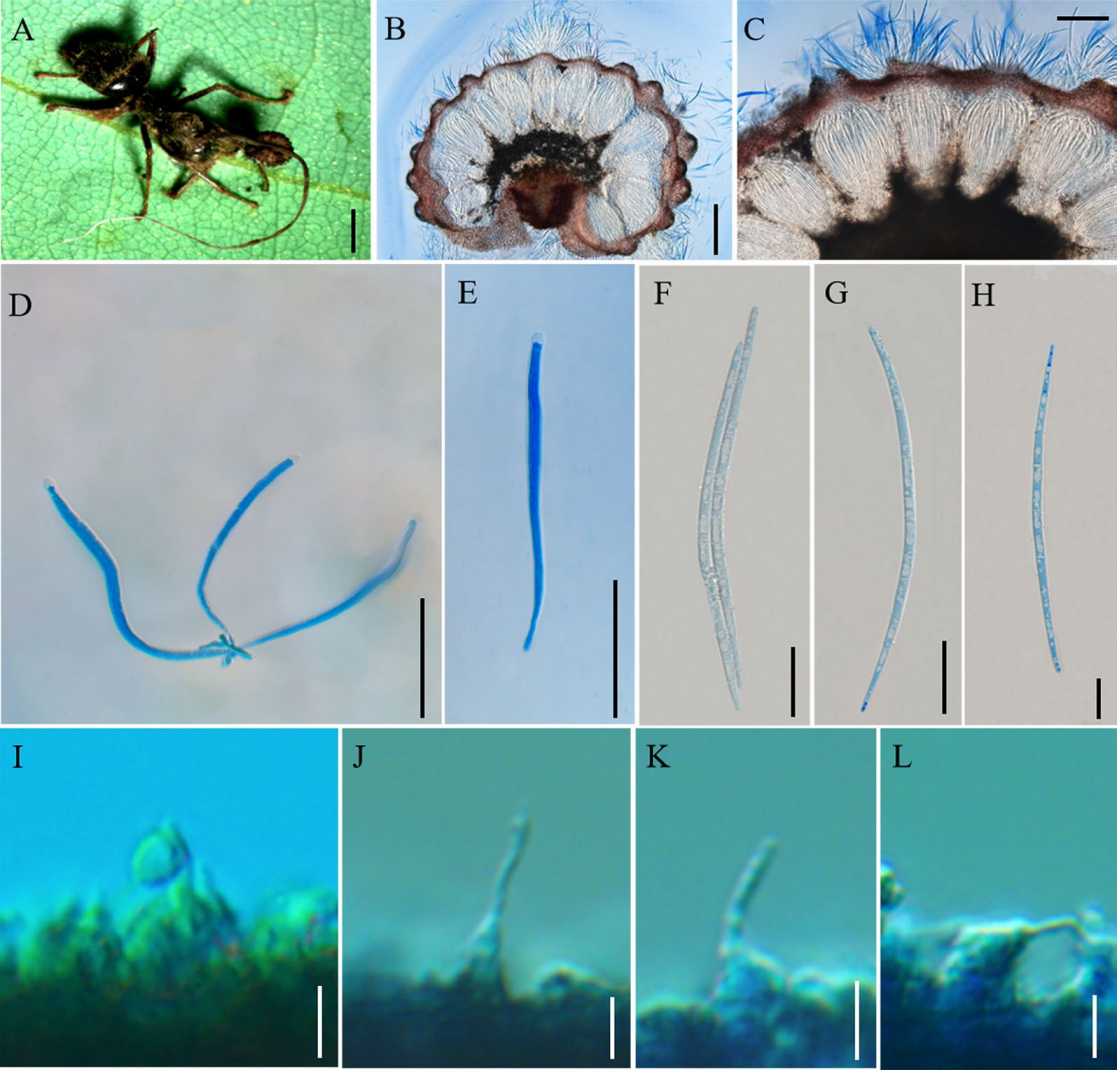
*Ophiocordyceps basiasca*. **A**: Infected *Camponotus* sp. was biting into a leaf of tree sapling. **B**, **C**: Cross-section of the ascoma showing the perithecial arrangement. **D**, **E**: Asci. **F–H**: Ascospores. **I–L**: Phialides and conidia. Scale bars: **A** = 2000 µm; **B** = 200 µm; **C** = 100 µm; **D**, **E** = 50 µm; **F**–**H** = 20 µm; **I–L** = 5 µm

Mycobank: MB 844353 (Fig. 6)

Etymology: The epithet refered to ascomata of lateral cushions produced from the basal of stromata.

*Diagnosis*: Similar to *O. contiispora* in conidia olivary, however, ascospores vermiform of *O. basiasca* was differed to *O. contiispora* (fusiform).

*Type*: **China:** Yunnan, Puer City, Sun River National Park. *Camponotus* sp. was infected and bited the middle vein of a leaf of tree seedling, 22°38′2″ N, 101°6′7″ E, alt. 1468 m, 19 Aug. 2020, Hong Yu bis (YHH 20190 – holotype preserved in the Yunnan Herbal Herbarium).

*Description*: **Sexual morph:** External mycelia produced from the sutures and joints, one stroma at the head of the ant, curved at the top, cylindrical, clavate, the base of stromata were dark brown, pale white at the top. Ascomata of lateral cushions produced from the basal of stromata, one ascoma was observed, spherical, brown, averaging 3 × 2 mm. Perithecia flask-shaped or ovoid, immersed to partially erumpent, with short, exposed neck or rounded ostiole, (195–) 202–242 (–248) × (92–) 102–149 μm. Asci cylindrical, hyaline, 8-spored, 96–188 (–212) × 4–9 (–10) μm. Ascus caps hemispherical, 3–5 µm high, 4–5 µm wide. Ascospores vermiform, hyaline, 4–5-septate, round to slightly tapered at the apex, 89–119 (–122) × 2–3 µm. **Asexual morph:** *Hirsutella* type-A only. Phialides lageniform, smooth, swollen base, tapering abruptly a neck, short, (8–) 10–23 (–26) × 1–5 µm. Conidia oviform, hyaline, smooth-walled, 1–4 × 1–2 µm.

Germination process: No ascospores examined from dried specimens.

*Host*: *Camponotus* sp. (Formicinae)

*Habitat*: Subtropical monsoon evergreen broad-leaf forest. *Camponotus* sp. was infected and bited into a leaf of tree seedling. It was collected from 1.5 m above the ground.

*Distribution*: China, Yunnan Province, Puer City

*Material examined*: **China:** Yunnan, Puer City, Sun River National Park. Infected ants were found biting into a leaf of tree seedling, 22°38′2″ N, 101°6′7″ E, alt. 1468 m, 19 August 2020, Hong Yu bis (YHH 20191).

Notes: Phylogenetic analyses showed that *O. basiasca* formed a separate clade in the *O. unilateralis* core clade; it was closed to *O. subtiliphialida* and *O. contiispora*, with statistical supported from BI posterior probabilities (PP = 100%) and ML bootstrap proportions (BP = 97%) (Fig. 1). *Ophiocordyceps basiasca* was similar to *O. subtiliphialida* and *O. contiispora* in lageniform phialides, olivary conidia. However, it differed from *O. subtiliphialida* and *O. contiispora* by vermiform ascospores, *Hirsutella* type-A.

### *Ophiocordyceps nuozhaduensis* Hong Yu bis & D.X. Tang, sp. nov.

**Fig. 7:**
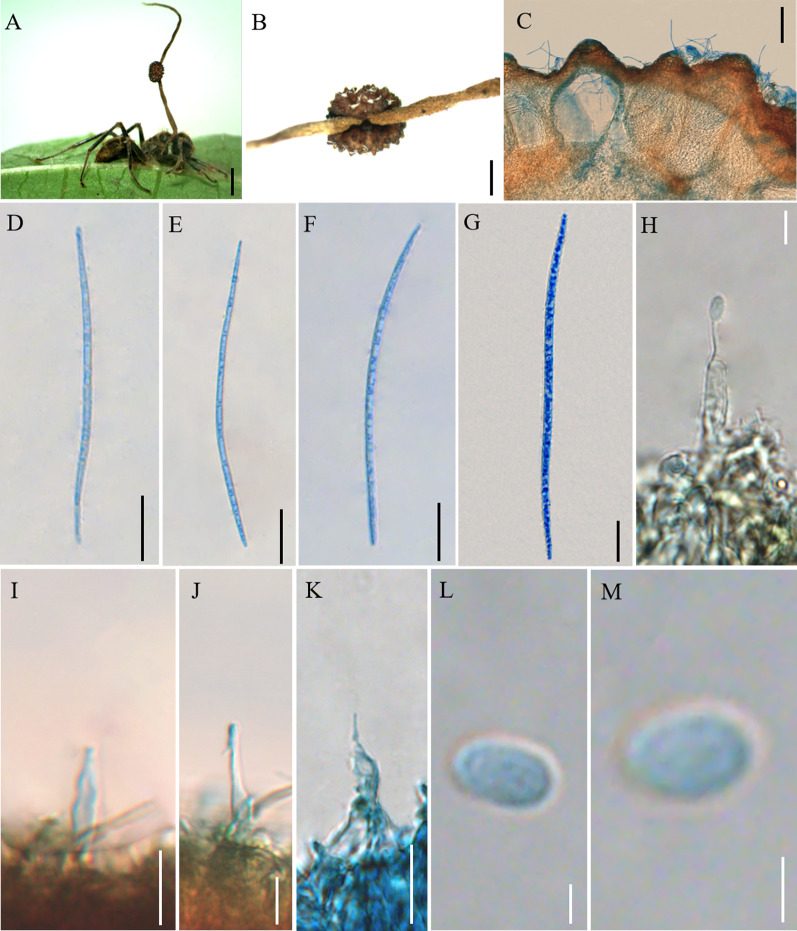
*Ophiocordyceps nuozhaduensis*. **A**: *Camponotus* sp. was infected and bited into a leaf of tree sapling. **B**: The ascoma was produced from the stroma. **C**: Cross-section of the ascoma showing the perithecial arrangement.** D–G**: Ascospores. **H**: Conidiogenous cells and conidia. **I–K**: Phialides. **L**, **M**: Conidia. Scale bars: **A** = 3000 µm; **B = **1000 µm; **C** = 100 µm; **D–G** = 20 µm; **H** = 5 µm; **I–K** = 10 µm; **L**, **M** = 2 µm

Mycobank: MB 844354 (Fig. 7)

Etymology: The epithet refered to the locality (Nuozhadu) where the holotype was collected.

*Diagnosis*: Similar to *O. camponoti-leonardi* in perithecia rounded ostiole, but *O. nuozhaduensis* differs by ellipsoidal or oviform conidia, smaller flask-shaped perithecia (215–285 × 128–172 μm).

*Type*: **China:** Yunnan, Puer City, Nuozhadu Nature Reserve. *Camponotus* sp. was infected and bited into a leaf of tree sapling, 22°38′27″ N, 100°29′53″ E, alt. 1107 m, 24 Aug. 2021, Hong Yu bis (YHH 20167 – holotype preserved in the Yunnan Herbal Herbarium).

*Description*: **Sexual morph:** External mycelia produced from sutures and joints of the ant. One stroma at the head of the ant, curved at the top, cylindrical, clavate, and dark brown at maturity. Fertile regions of lateral cushions produced from the middle of stromata, one ascoma was observed, spherical, brown, averaging 2.4 × 1.6 mm. Perithecia flask-shaped, immersed to partially erumpent, with short, exposed neck or rounded ostiole, (215–) 222–274 (–285) × (128–) 153–159 (–172) μm. Asci were not observed. Ascospores vermiform, hyaline, 7–13-septate, round to slightly tapered at the apex, 91–126 (–132) × 2–5 µm. **Asexual morph:** *Hirsutella* type-A present on the stroma and the legs. Phialides cylindrical or lageniform, smooth, swollen base, tapering abruptly a neck, short, 6–22 (–22) × 2–4 µm. Conidia ellipsoidal or oviform, 2–5 × 2–3 µm.

Germination process: No germination examined because the specimens were dried.

*Host*: *Camponotus* sp. (Formicinae)

*Habitat*: Subtropical monsoon evergreen broad-leaf forest. *Camponotus* sp. was infected and bited into a leaf of tree sapling. Always at lower heights, collected from 25 to 50 cm above the ground.

*Distribution*: China, Yunnan Province, Puer City.

*Material examined*: **China:** Yunnan, Puer City, Nuozhadu Nature Reserve. Infected ants were found biting a leaf of tree seedling, 22°38′27″ N, 100°29′53″ E, alt. 1107 m, 24 Aug. 2021, Hong Yu bis (YHH 20168, YHH 20169).

Notes: Phylogenetically, this species was closed to *O. camponoti-leonardi*, was clustered in the *O. unilateralis* core clade, with high statistical supported by BI (PP = 98%) and ML (BP = 100%) (Fig. 1). It was similar to sister *O. camponoti-leonardi* in rounded ostiole perithecia. However, it differed from *O. camponoti-leonardi* in vermiform ascospores, ellipsoidal or oviform conidia.

### *Ophiocordyceps contiispora* Hong Yu bis & D.X. Tang, sp. nov.

**Fig. 8:**
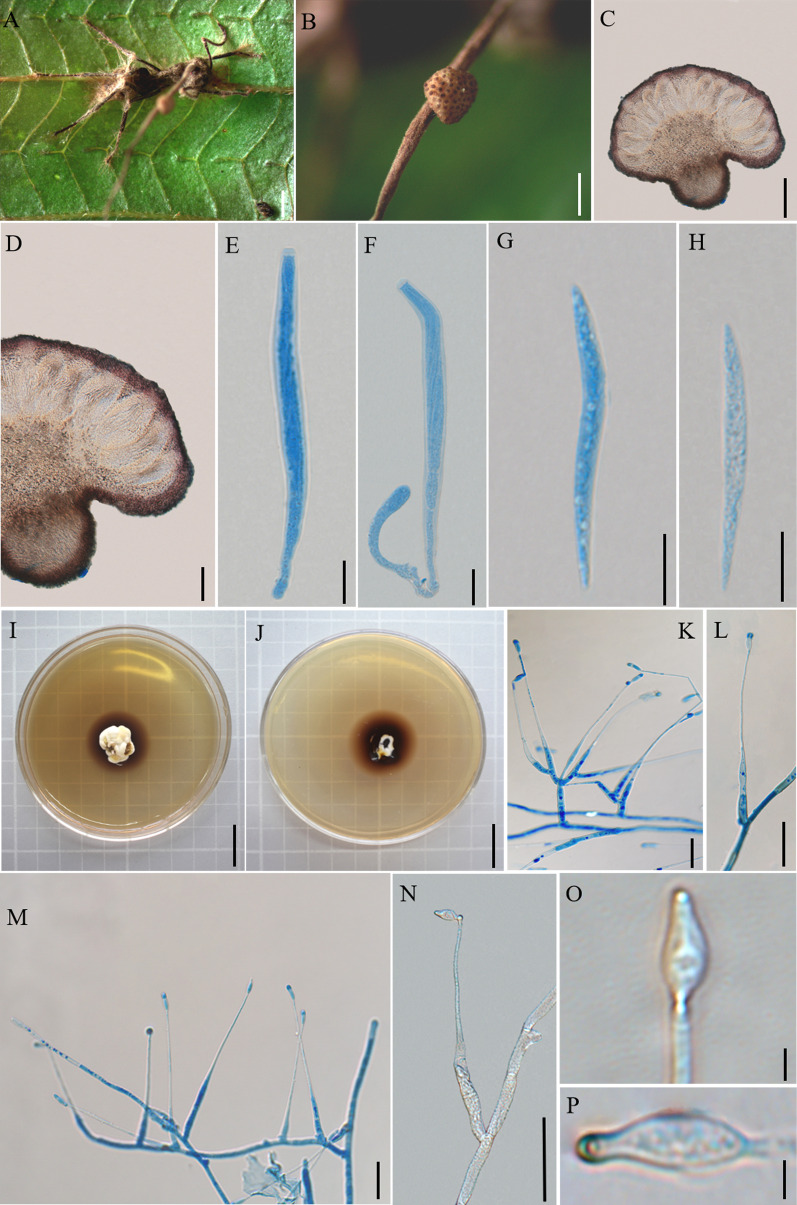
*Ophiocordyceps contiispora*. **A**: Infected *Camponotus* sp. was biting into a leaf of epiphytes. **B**: Close-up of the ascoma. **C**, **D**: Cross-section of the ascoma showing the perithecial arrangement. **E**, **F**: Asci. **G**, **H**: Ascospores. **I**, **J**: Colonies on PDA medium. **K**: Conidiophores and phialides. **L–N**: Conidiogenous cells and conidia. **O**,** P**: Conidia. Scale bars: **A** = 1000 µm; **B** = 500 µm; **C** = 200 µm; **D** = 100 µm; **E**, **F** = 20 µm; **G, H** = 10 µm;** I**, **J** = 2 cm; **K–N** = 20 µm; **O, P** = 2 µm

Mycobank: MB 844355 (Fig. 8)

Etymology: The epithet refered to the top of conidia having a protuberance like a spear.

*Diagnosis*: Similar to *O. subtiliphialida* in the top of conidia has a protuberance, but the protuberance of *O. contiispora* was more prominent and the width of conidia was smaller (4–6 × 1–2 μm) than *O. subtiliphialida* (6–10 × 3–6 μm).

*Type*: **China:** Yunnan, Mengla County, Mohan Town, Xinming Village. *Camponotus* sp. was infected and bited into a leaf of epiphytes, 21°9′35″ N, 101°45′49″ E, alt. 1173 m, 2 Oct. 2019, Hong Yu bis (YHH 20144 – holotype preserved in the Yunnan Herbal Herbarium; living culture YFCC 9027 – ex-holotype stored in Yunnan Fungal Culture Collection).

*Description*: **Sexual morph:** External mycelia produced dense from the joints, covering the host body, sparsely when touching the substrate. Stromata single, produced from dorsal pronotum of the ant, cylindrical, clavate, brown at maturity. Fertile part of lateral cushions produced from stromata, one ascoma was observed, disc-shaped, brown, averaging 1.3–1.8 × 1–1.5 mm. Perithecia flask-shaped, immersed to partially erumpent, with short, exposed ostiole, (146–) 158–212 (–224) × 69–122 μm. Asci cylindrical, hyaline, curved, 8-spored, (74–) 89–130 (–134) × 4–9 μm. Ascus caps hemispherical or square, small, 1–3 µm high, 3–5 µm wide. Ascospores fusiform, hyaline, no obvious separation, occasionally curved, round to slightly tapered at the apex, (29–) 38–48 (–62) × 2–4 µm. **Asexual morph:** Colonies on PDA medium slow-growing, 28–30 mm diameter in 30 days at 25 °C, milky white to light brown, raising cottony-shaped mycelia density, protuberant mycelia at the centrum, reverse light brown to dark brown. Hyphae immersed in the medium, milky white, branched, septate, smooth-walled, hyaline. *Hirsutella* type-C only. Conidiophores rare, cylindrical, produced from the hyphae, septate, short, 11–12 × 3–4 µm. Conidiogenous cells monophialidic or rarely polyphialidic, forming on side hyphae or conidiophores, smooth, swollen base, lageniform, tapering gradually a long neck, straight, (42–) 57–92 (–97) × 1–4 µm. Conidia olivary or flask-shaped, hyaline, the top of conidia has a protuberance like a spear, smooth-walled, 4–6 × 1–2 µm.

Germination process: No germination observed from dried specimens.

*Host*: *Camponotus* sp. (Formicinae)

*Habitat*: Rainforest and subtropical monsoon evergreen broad-leaf forest. *Camponotus* sp. was infected and bited into a leaf of epiphytes. Dying in an elevated position, collected from 1 to 2 m above the ground.

*Distribution*: China, Yunnan Province, Puer City and Jinghong City.

*Material examined*: **China:** Yunnan, Mengla County, Mohan Town, Xinming Village. *Camponotus* sp. was infected and bited into a leaf of epiphytes, 22°21′20″ N, 101°69′01″ E, alt. 865 m, 3 Oct. 2019, D.X. Tang (YHH 20145; living culture YFCC 9026). Other specimens were collected from China, Yunnan Province, Puer City, Sun River National Park. Infected ants were found biting into a leaf of tree sapling, 22°38′2″ N, 101°6′7″ E, alt. 1468 m, 19 Aug. 2020, Hong Yu bis (living culture YFCC 9025).

Notes: *Ophiocordyceps contiispora* was phylogenetically sister to *O. basiasca* with high statistical supported by BP = 100% and PP = 100%. It was similar to *O. basiasca* in flask-shaped perithecia, cylindrical asci, lageniform phialides. However, it differed from *O. basiasca* by fusiform ascospores, producing *Hirsutella* type-C.

## Discussion

Many phylogenetic classifications have been undertaken for *O. unilateralis *sensu lato (Evans et al. 2011a; kobmoo et al. 2012, 2015; Araújo et al. 2018; Wei et al. 2020), these groups have been continuously supplemented and improved based on morphology, molecular phylogeny and ecology. This study focused on the phylogenetic investigation of *O. unilateralis *sensu lato  species collected from Yunnan Province, China. The phylogenetic tree showed that six new species were clustered in the *O. unilateralis* core clade of *Hirsutella* (Fig. 1). Four species (*O. contiispora*, *O. basiasca*, *O. subtiliphialida*, and *O. acroasca*) were formed a sister lineage with *O. septa*. In addition, *O. bifertilis* formed a sister lineage with *O. satoi* and *O. naomipierceae*, and *O. nuozhaduensis* also formed a sister lineage with *O. camponoti-leonardi*. The phylogenetic framework was consistent with previous studies (Kobmoo et al. 2012; Crous et al. 2016; Araújo et al. 2018; Wei et al. 2020). However, some species were lower support for topologies, including *O. camponoti-leonardi*, *O. polyrhachis-furcata*, *O. nuozhaduensis*, *O. ootakii* and *O. nooreniae*. The reason might be that a few genes were used for *O. camponoti-leonardi* and *O. polyrhachis-furcata*. Both phylogenetic analysis and morphological characters supported that the six fungi were distinctive in the core clade of *O. unilateralis*. Six new species were proposed to be located in the *O. unilateralis* core clade of the *Hirsutella* clade within *Ophiocordyceps*.

The *O. unilateralis* complex was composed of *O. unilateralis* core clade, *O. oecophyllae* clade, *O. kniphofioides* sub-clades (Araújo et al. 2018). Many species were described in the *O. unilateralis* core clade, with most of the hosts being *Camponotus* and *Polyrhachis*. Species within the *O. unilateralis* core clade shared many macro-morphological characteristics that made them easily recognized in this study, such as stromata, ascomata, type of host, and location of host attachment. *Ophiocordyceps unilateralis* complex species commonly bitten and attached leaves, spines, epiphites, saplings, moss, twing in a "death grip" (Evans et al. 2011b, 2018; Hughes et al. 2011; Kepler et al. 2011; Luangsa-ard et al. 2011; Kobmoo et al. 2012, 2015; Araújo et al. 2015, 2018; Crous et al. 2016), with dying in an elevated position, from 0.25 to 2 m or higher above the ground. Fewer *O. unilateralis* complex species did not have biting and grasping behavior, such as *O. tianshanensis* (Wei et al. 2020). These species, i.e., *O. acroasca*, *O. basiasca*, *O. bifertilis*, *O. contiispora*, *O. nuozhaduensis*, and *O. subtiliphialida*, died by biting onto the middle vein of a sapling, Pteridophyta, Gramineae, and epiphytes in death position, and at an elevated position, from 0.25 to 2 m above the ground, with results being consistent with previous work (Andersen et al. 2009; Araújo et al. 2018). Species of the *O. unilateralis* complex have been investigated at the same site in this survey for two years. It was found that the height at which the host died on vegetation from the ground appeared to be affected by climate, such as rainfall, humidity, temperature, etc.. The death position of species in the *O. unilateralis* complex was 0.5 to 1 m or higher above the ground in the first year, while these species died 0.25 m or less above the ground in the second year. This adaption might occupy a niche and provide for effective spores dispersal (Andersen et al. 2009; Hughes et al. 2011).

The ant manipulation behavior of the *O. unilateralis* complex occurred only in host-specific species, especially those entomopathogenic fungi that were parasitic on the ants of *Camponotus* (Evans et al. 2011b; de Bekker et al. 2014; Araújo et al. 2018; Sakolrak et al. 2018). Crous et al. (2016) reported that *O. nooreniae* infected two host species of *Polyrhachis* (*Polyrhachis* cf. hookeri and *Polyrhachis lydiae*). Our survey also found that a fungus infected multiple hosts of *Polyrhachis*, and behavior manipulation of the ant almost tended to be consistent, such as the host *Polyrhachis* sp.1 and *Polyrhachis* sp.2 were infected by *O. bifertilis*. The majority of ant pathogenic fungi parasitic on the host of the genus *Polyrhachis* were reported from Southeast Asia, and some species found in Australia (Crous et al. 2016; Araújo et al. 2018). Pathogenic fungi infecting *Polyrhachis* ants, such as *O. bifertilis*, often induced the host to bite onto the main vein of a Pteridophyta leaf. It was similar to *O. naomipierceae*, *O. ootakii*, *O. polyrhachis-furca*, *O. nooreniae* and *O. satoi* in phylogeny, habitat, biting and attachment behaviour. The phylogenetic tree of the host ants also indicated that they were closely related species (Fig. 2). This evidence showed that the pathogenic fungi and the host ants were closely related in genetic evolution, and that the diversity of the host might affect the diversity of the pathogenic fungus. *Polyrhachis* was the second most species-rich genus in Formicinae, currently comprising 706 valid species (http://antcat.org/2022). *Polyrhachis* originated in Southeast Asia, and dispersed out of Southeast Asia to Australia (Mezger and Moreau 2015). They were widely distributed, ranging from tropical regions in Africa and Asia to Australia and a few Pacific islands. The highest species richness and diversity were in China and Australia. Currently, at least five pathogenic fungi of the host *Polyrhachis* had been reported in Australia and Southeast Asia. There might exist many pathogenic fungi hosted by *Polyrhachis* ants to be discovered worldwide, especially in China, Southeast Asia, and Austrilia.

Parasite manipulation of host behavior was an active research topics in various fields (Evans et al. 2011b, 2018; Hughes et al. 2011; Kepler et al. 2011; Luangsa-ard et al. 2011; Kobmoo et al. 2012, 2015; Araújo et al. 2015, 2018; Crous et al. 2016; de Bekker et al. 2018; Will et al. 2020). Multiple reports indicated that manipulation of ant behavior was host-specific (de Bekker et al. 2014, 2018). Host-specific fungal species seemed to be associated with each ant species, leading to the "one ant, one fungus", and the host identity used as criteria for fungal species identification (Evans et al. 2011b; Kobmoo et al. 2012; Araújo et al. 2015, 2018). Population genomics also supported the host-specificity in ant pathogenic fungi by Kobmoo et al. (2019). In this work, the result suggested that multiple ant pathogenic fungi, including *O. acroasca*, *O. basiasca*, *O. contiispora*, *O. subtiliphialida* (Fig. 2), infecting the same host *Camponotus* sp.. Interestingly, Kobmoo et al. (2019) revealed that genetic clusters in ant pathogenic fungi sharing the same host. This study supports previous studies that the same host of *Camponotus* can be infected by different ant pathogenic fungi, while the ant pathogenic fungi of *Polyrhachis* can infect multiple hosts at the same time. It is not known that an ant fungus infects multiple hosts of the genus *Camponotus* at the same time. *Ophiocordyceps* *unilateralis* complex species were composed of distinct evolutionary species leads to a global diversity of the ant pathogenic fungi (Kobmoo et al. 2012, 2015; Araújo et al. 2015, 2018; Crous et al. 2016). *Camponotus* was the most species-rich genus in Formicinae, currently comprising 1087 valid species (http://antcat.org/2022). *Camponotus* ants were distributed in the terrestrial environment worldwide*.* However, up to now, less than 30 pathogenic fungi have been reported to parasitize *Camponotus* ants, and some ant pathogenic fungi tend to between sharing the same microhabitat and niche overlap, which might lead to a diversity of the ant pathogenic fungi.

In recent decades, a large amount of research has been conducted to discuss how many fungi exist in the world (Weir and Hammond 1997; Hawksworth 2001; Hawksworth and Lücking 2017). Through the continuous efforts of scientists, the original estimate of 1.5 million (Hawksworth 2001) fungi has changed to 2.2 to 3.8 million fungi (Hawksworth and Lücking 2017). However, relatively few studies have discussed the number of entomopathogenic fungi worldwide. One significant study on host-specificity by Weir and Hammond (1997) in relation to insects was that on the Laboulbeniales on beetles. These studies suggest a beetle (Coleoptera): fungus ratio of 1.68–2: 1. Araújo and Hughes (2019) research shows that zombie-ant fungal lineage likely arose from an ancestor that infected beetle (Coleoptera) larvae. At present, it has been reported that seven genera in the family Formicidae were infected by the *O. unilateralis* complex species, including *Camponotus*, *Cephalotes*, *Daceton*, *Dolichoderus*, *Oecophylla*, *Paraponera* and *Polyrhachis* (Evans and Sampson 1982, Kepler et al. 2011, Evans et al. 2011b, Kobmoo et al. 2012, Luangsa ard et al. 2011, Araújo et al. 2015, Kobmoo et al. 2015, Crous et al. 2016, Araújo et al. 2018, Evans et al. al. 2018, Wei et al. 2020). There were 2046 valid species in seven genera of Formicidae (excluding valid subspecies) (https://antcat.org/2022). If all entomopathogenic fungi accord with the beetle (Coleoptera): fungus ratio of 1.68–2: 1 by Weir and Hammond (1997), then there may be 1217–1023 species of entomopathogenic fungi in the world that can infect ants of seven genera in Formicidae, including the *O. unilateralis* complex.

Morphological characters were diverse for *O. unilateralis *sensu lato species. Most of the morphological features of *O. unilateralis *sensu lato species included cylindrical and clavate stromata that arose from the dorsal pronotum of the host, at least one ascoma that grew from lateral cushions of stromata. Some species produced multiple stromata, such as *O. camponoti-indiani*, *O. halabalaensis*, *O. satoi* (Araújo et al. 2015, 2018). Similar results were obtained in this study, the species, *O. bifertilis*, two stromata produced from the head of *Polyrhachis* sp.1 and *Polyrhachis* sp.2, resulting in two ascomata from stromata. Ascomata of *O. unilateralis *sensu lato were usually characterized by hemispherical, disc-shaped, spherical, one to multiple. All species in this group produced ascospores that were not disarticulate into part spores, and the shape includes vermiform, cylindrical, lanceolate, and fusiform. These shapes might to better dispersal for their spores.

Most species formed an asexual morph characterized by *Hirsutella* type-A phialides, tapering to a long neck and bearing a single conidium at their apices. There were also two types of asexual morphs, i.e., *Hirsutella* type-B and *Hirsutella* type-C. Most species produced phialides along stromata, legs and joints. The phialides of these species, such as *O. basiasca* (*Hirsutella* type-A), *O. bifertilis* (*Hirsutella* type-A), *O. nuozhaduensis* (*Hirsutella* type-A), were also observed from stromata, legs and joints. However, their phialides were shorter than *O. acroasca* (*Hirsutella* type-A and *Hirsutella* type-C), *O. subtiliphialida* (*Hirsutella* type-C) and *O. contiispora* (*Hirsutella* type-C) (Table 4). The phialides of *O. acroasca*, *O. subtiliphialida*, *O. contiispora* were produced from pure culture. This structure, rarely polyphialidic and conidiophores, were observed in the species of *O. subtiliphialida*, *O. contiispora*. *Ophiocordyceps subtiliphialida* and *O. contiispora* were only observed in *Hirsutella* type-C, and *Hirsutella* A-type was not been observed. The same result was also reported in Araújo et al. (2018). Unfortunately, the phialides produced from pure cultures and specimens were not compared, as specimens were used to isolate strains, or were dried, or made into permanent specimens. Conidia were diverse in *O. acroasca* (limoniform), *O. basiasca* (oviform), *O. nuozhaduensis* (ellipsoidal or oviform), *O. subtiliphialida* (olivary) and *O. contiispora* (olivary or flask-shaped) (Table 4). In addition, characteristics of the living cultures were introduced in the present work more than in previous studies (Kobmoo et al. 2012; Crous et al. 2016; Araújo et al. 2018; Wei et al. 2020). They were slow-growing, hard, light brown to dark brown in color, and produced pigment. This work has provided a method (see materials and methods for details) for obtaining living cultures of *O. unilateralis* complex species and asexual morph based on pure culture, which is of real value for further studies of *O. unilateralis* complex species in the future.

## Conclusions

Six zombie-ant fungi were described from Yunnan Province, China. These novel species of *Ophiocordyceps* with hirsutella-like asexual morphs exclusively infecting ants were well supported based on molecular phylogenetic data and morphological evidence. This work proposes that the same host of *Camponotus* can be infected by multiple ant pathogenic fungi, while multiple species of *Polyrhachis* can be infected by the same pathogenic fungi at the same time. This study provides six new taxa support to explore the evolutionary relationship between the host and the fungus, and provides novel insights into the morphology, parasitism, distribution and ecology of *O. unilateralis *sensu lato within *Ophiocordyceps*. It has provided a method to obtain living cultures of the *O. unilateralis* complex and asexual morphs based on pure culture, which is of great value for further future studies of zombie-ant fungi.

## Key to *Ophiocordyceps unilateralis* complex species worldwide

1a. On host *Camponotus*…………………………………………………………………………………………………………...2

1b. On host *Cephalotes*…………………………………………………………………………………….*Ophiocordyceps kniphofioides*

1c. On host *Daceton*………………… …………………………………………………………………..*Ophiocordyceps daceti*

1d. On host *Dolichoderus*………………………………………………………………………………..*Ophiocordyceps monacidis*

1e. On host *Oecophylla*…………………………………………………………………………………..*Ophiocordyceps oecophyllae*

1f. On host *Paraponera*…………………………………………………………………………………..*Ophiocordyceps ponerinarum*

1 g. On host *Polyrhachis*………………………………………...............................................................................................14

2a. The host without a biting behavior…………………………………………………………………...*Ophiocordyceps tianshanensis*

2b. The host with biting behavior………………………………………...................................................................................3

3a. Death position of the host was biting leaf………………………………….................................................................4

3b. Death position of the host was biting twing………………………………..............................................................12

4a. Ascospores not obvious separation………………............................................................................*Ophiocordyceps contiispora*

4b. Ascospores obvious separation………………………………………..................................................................................5

5a. The widest ascospore was not more than 3 μm……………………………………..........................................................6

5b. The widest ascospore was more than 3 μm…………………………………................................................................7

6a. Ascospores 83–108 × 2–3 μm, perithecia 247–296 × 176–225 μm, asci 131–172 × 5–8 μm………...*Ophiocordyceps acroasca*

6b. Ascospores 89–119 × 2–3 μm, perithecia 202–242 × 102–149 μm, asci 96–188 × 4–9 μm………….*Ophiocordyceps basiasca*

6c. Ascospores 110–125 × 2–3 μm, perithecia 400–430 × 200–230 μm, asci 130–175 × 7–8 μm……….*Ophiocordyceps camponoti-leonardi*

6d. Ascospores 75–85 × 2–3 μm, perithecia 280–320 × 160–180 μm, asci 80–160 × 6–7 μm…………...*Ophiocordyceps camponoti-saundersi*

6e. Ascospores 200–215 × 2–3 μm, perithecia 325–500 × 275–300 μm, asci 200–340 × 7–10 μm……...*Ophiocordyceps rami*

6f. Ascospores 80–85 × 3 μm, perithecia 240–280 × 100–150 μm, asci 110–140 × 6–6.5 μm…………...*Ophiocordyceps camponoti-atricipis*

6g. Ascospores 75–90 × 3 μm, perithecia 200–230 × 135–165 μm, asci 110–130 × 8–9 μm…………….*Ophiocordyceps camponoti-femorati*

6h. Ascospores 80–95 × 2–3 μm, perithecia 175–260 × 100–130 μm, asci 120–160 × 8–10 μm………..*Ophiocordyceps camponoti-rufipedis*

6i. Ascospores 120–140 × 3 μm, perithecia 225–230 × 135 μm, asci 150–160 × 8–9 μm……………….*Ophiocordyceps camponoti-sexguttati*

6j. Ascospores 75–85 × 2–2.5 μm, perithecia 200–250 × 140–160 μm, asci 95–125 × 6–8 μm………....*Ophiocordyceps unilateralis*

7a. Ascospores vermiform………………………………………...........................................................................................8

7b. Ascospores cylindrical…………………………………..............................................................................................10

7c. Ascospores filiform………………...................................................................................................*Ophiocordyceps camponoti-novogranadensis*

7d. Ascospores lanceolate…………………………………………...............................................................................................11

8a. Ascospores the longest was not more than 85 μm………………......................................................*Ophiocordyceps camponoti-chartificis*

8b. Ascospores the longest was more than 85 μm………………………………............................................................9

9a. Ascospores 91–126 × 2–5 μm, perithecia 222–274 × 153–159 μm……………….............................*Ophiocordyceps nuozhaduensis*

9b. Ascospores 90–105 × 3–4 μm, perithecia 200–240 × 100–150 μm……………….............................*Ophiocordyceps camponoti-nidulantis*

9c. Ascospores 90–120 × 4 μm, perithecia 220–250 × 100–165 μm………………..................................*Ophiocordyceps camponoti-renggeri*

10a. Ascospores 80–100 × 5 μm, distributed in Colombia………………................................................*Ophiocordyceps albacongiuae*

10b. Ascospores 60–75 × 3–5 μm, distributed in Thailand………………................................................*Ophiocordyceps halabalaensis*

10c. Ascospores 135–175 × 4–5 μm, distributed in Brazil……………….................................................*Ophiocordyceps camponoti-balzani*

10d. Ascospores 70–75 × 4.5–5 μm, distributed in Brazil………………..................................................*Ophiocordyceps camponoti-bispinosi*

10e. Ascospores 75–90 × 4–5 μm, distributed in USA……………….......................................................*Ophiocordyceps camponoti-floridani*

10f. Ascospores 75–85 × 4–5 μm, distributed in Brazi…………………...................................................*Ophiocordyceps camponoti-hippocrepidis*

10g. Ascospores 75 × 4.5 μm, distributed in Brazil………………............................................................*Ophiocordyceps camponoti-indiani*

10h. Ascospores 170–210 × 4–5 μm, distributed in Brazil…………………..............................................*Ophiocordyceps camponoti-melanotici*

11a. Ascospores 45–50 × 6–8 μm, distributed in Thailand…………………..............................................*Ophiocordyceps septa*

11b. Ascospores 52–72 × 5–8 μm, distributed in China………………......................................................*Ophiocordyceps subtiliphialida*

12a. Ascospores cylindrical………………………………………………................................................................................................13

12b. Ascospores filiform………………....................................................................................................*Ophiocordyceps pulvinata*

13a. Two types of *Hirsutella* asexual morph………………......................................................................*Ophiocordyceps kimflemingiae*

13b. One types of *Hirsutella* asexual morph………………......................................................................*Ophiocordyceps blakebarnesii*

14a. Biting leaf……………………………………..................................................................................................................15

14b. Biting twing………………...............................................................................................................*Ophiocordyceps satoi*

15a. *Paraisaria*-like phialides………………............................................................................................*Ophiocordyceps naomipierceae*

15b. *Hirsutella*-like phialides………………………………….............................................................................................16

16a. Two types of *Hirsutella* asexual morph……………….......................................................................*Ophiocordyceps nooreniae*

16b. One types of *Hirsutella* asexual morph…………………………….......................................................................17

17a. Phialides 9–24 × 2–4 μm, distributed in China………………............................................................*Ophiocordyceps bifertilis*

17b. Phialides 6–8 × 3–4 μm, distributed in Japan………………..............................................................*Ophiocordyceps ootakii*

17c. Phialides 30 × 2–3 μm, distributed in Thailand………………...........................................................*Ophiocordyceps polyrhachis-furca*

## Data Availability

All sequence data generated for this work can be accessed via GenBank: https://www.ncbi.nlm.nih.gov/genbank/. All alignments for phylogenetic analyses were deposited in TreeBASE (http://www.treebase.org; the following links were available: http://purl.org/phylo/treebase/phylows/study/TB2:S29994?x-access-code=8e258e97fca38d4f834975a2fefb47a1&format=html.)

## Abbreviations

BI: Bayesian inference
BP: Bayesian posterior probability
bp: Base pair
CTAB: Cetyl-trimethyl-ammonium bromide
DNA: Deoxyribonucleic acid
ML: Maximum likelihood
SSU: The nuclear ribosomal small subunit
LSU: The nuclear ribosomal large subunit
PCR: Polymerase chain reaction
PP: Posterior probabilities
PDA: Potato dextrose agar
*RPB1*: The largest subunits of RNA polymerase II
*RPB2*: The second largest subunits of RNA polymerase II
*TEF*: The translation elongation factor 1α
